# Process evaluation of a basic life support educational intervention (FirstCPR cluster randomised study) delivered at community organisations in New South Wales, Australia

**DOI:** 10.1136/bmjopen-2025-113343

**Published:** 2026-04-07

**Authors:** Sonali Munot, Adrian Bauman, Janet Bray, Julie Redfern, Blake Angell, Diane Kancijanic, Zoe Rock, Christopher Semsarian, Garry Jennings, Andrew Coggins, Alan Robert Denniss, Clara Chow

**Affiliations:** 1 The University of Sydney Westmead Applied Research Centre, Westmead, New South Wales, Australia; 2 The University of Sydney School of Public Health, Sydney, New South Wales, Australia; 3 Monash University School of Public Health and Preventive Medicine, Melbourne, Victoria, Australia; 4 Bond University Institute for Evidence-Based Healthcare, Robina, Queensland, Australia; 5 University of New South Wales, Sydney, New South Wales, Australia; 6 The University of Sydney, Sydney, New South Wales, Australia; 7 The University of Sydney Charles Perkins Centre, Sydney, New South Wales, Australia; 8 Department of Emergency Medicine, Westmead Hospital, Westmead, New South Wales, Australia; 9 Department of Cardiology, Westmead Hospital, Westmead, New South Wales, Australia

**Keywords:** Out-of-Hospital Cardiac Arrest, Cardiopulmonary Resuscitation, Health Education, PUBLIC HEALTH

## Abstract

**Background:**

The FirstCPR cluster randomised trial delivered multimodal basic life support (BLS) learning opportunities to community organisations. An a priori process evaluation examined intervention implementation, including participation, reach, uptake and member engagement.

**Methods:**

The study used a multimethod process evaluation. Data were collected via semistructured interviews, focus group discussions, participant surveys, study records, web analytics and in-field observations. These sources captured participation patterns and implementation measures (delivery, reach, uptake and engagement: opt-in to digital messages and attendance at training sessions), as well as reasons for refusals and withdrawals. Qualitative data were analysed thematically and organised using the *UK Medical Research Council process-evaluation framework*. Qualitative and quantitative data were analysed separately and subsequently interpreted collectively to contextualise implementation patterns and identify barriers and enablers that influenced trial successes and failures.

**Results:**

Intervention uptake and engagement varied significantly across organisations, with greater success observed in social and faith-based groups. Of the 82 intervention clusters, 78 (95%) received intervention materials; 74 (90%) engaged in at least one activity and 15 (18%) engaged in all activities. Participation was primarily driven by the organisation’s leadership interest and support in providing BLS training to members, and by the time available to facilitate intervention activities. The presence of a dedicated liaison/champion emerged as the most critical enabler of member engagement and implementation. Feedback recommended concise, simple and culturally tailored modules, with practical components delivered in shorter, convenient sessions. Intervention delivery was affected by contextual challenges, including COVID-19 disruptions that limited in-field recruitment and group activities.

**Conclusions:**

Process evaluation can strengthen community-based interventions by identifying mechanisms and contextual factors that shape implementation and engagement. Partnering with social and faith-based organisations may be an effective approach to disseminating educational programmes such as life-saving skills to lay communities. Minimising research burden and ensuring organisational leadership support may improve participation while brief, practical and culturally tailored training may enhance engagement.

**Trial registration number:**

ACTRN12621000367842.

**STRENGTHS AND LIMITATIONS OF THIS STUDY**

- A multimethods process evaluation enabled a nuanced understanding of intervention implementation across diverse community contexts.
- The use of multiple data sources enabled the capture of diverse perspectives from participants and facilitators.
- Real-world delivery provided practical insights into implementation and community engagement, although engagement could not be fully quantified across all dissemination channels.
- The multimodal intervention design, including digital components, supported access and some continuity of delivery when COVID-19 restricted in-person activities.
- Limited input from non-participants reduced insight into engagement barriers, although diverse stakeholder perspectives were captured from participating organisations and community members.

## Introduction

 Bystander cardiopulmonary resuscitation (CPR) and defibrillation before ambulance arrival significantly improve out-of-hospital cardiac arrest (OHCA) survival.^1^ However, limited knowledge and confidence in basic life support (BLS) remain a key barrier to bystander response.^2 3^ Currently, in Australia, BLS training is mainly classroom-based, fee-based, accessed for professional reasons,^4^ monolingual^5^ and often inaccessible to certain groups.^6 7^ There is international consensus on the need to implement and evaluate novel strategies to improve community-wide awareness and education.^8^

The FirstCPR cluster randomised controlled trial examined the implementation of BLS education to members of the community via their social, faith-based and sports organisations (clusters).^9^ The FirstCPR intervention package was designed to be delivered over 12 months with maintenance of high visibility and providing access to both digital and in-person learning opportunities for broader reach.^9 10^ At the end of the trial, a greater percentage of members from intervention clusters reported they were ‘trained and willing to perform CPR on a stranger’ compared with control members (64% vs 47%, OR 2.22 (95% CI 1.50 to 3.30).^11^ Rates of confidence and willingness to use an automated external defibrillator (AED) followed similar trends.

A process evaluation provides complementary information to contextualise trial results and can contribute to the subsequent translation of findings to a broader context.^12^,^14^ The goal of this paper is to report on the prespecified process evaluation conducted alongside the FirstCPR trial. The aims were to examine (1) organisation enrolment and factors that influenced study participation; and (2) intervention implementation, including factors that influenced reach, uptake and engagement.^15 16^

## Methods

### Study design

**Figure 1:**
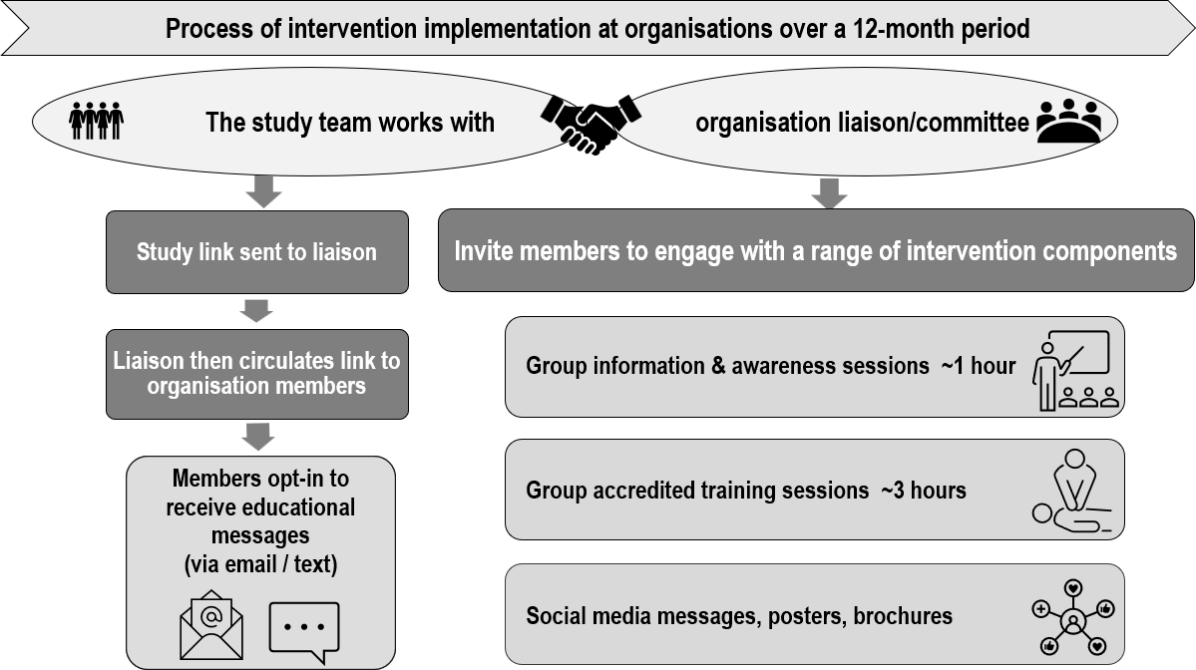
Process of intervention implementation at enrolled organisations.

A multimethod process evaluation was conducted alongside the implementation of the FirstCPR trial, with quantitative and qualitative data collected during recruitment, as well as during and after implementation of the intervention.^17^ The FirstCPR trial is described elsewhere, and the intervention components and implementation process are summarised in figure 1 and online supplemental figure S1.^11^ Community organisations (social, faith-based, sports groups, workplaces) in the targeted areas were searched online, via local councils and community networks, and invited to participate in the study.^9^ Enrolment and randomisation took place from 20 April to 20 December 2021, with staggered intervention implementation at participating sites.

Community organisation committees, including presidents or secretaries, were the decision-makers on study enrolment and acted as committee liaisons. The study team collaborated with liaisons to deliver intervention components to organisation members (figure 1). Throughout the study, committee liaisons served as the conduit to reach organisation members and facilitated the implementation of various intervention components within organisations.

### Data sources

Process evaluation measures were planned a priori to explore the intervention strategy, design and delivery, potential mechanisms of action, and any extraneous and contextual factors that influenced participation and uptake.^16^ A multimethod approach was used, with data collected via study team observations and field notes, web analytics, surveys, semistructured interviews and focus group discussions.^18^ Quantitative and qualitative datasets were analysed separately. Findings from the various data sources were subsequently considered together during interpretation to provide a comprehensive understanding of intervention delivery, programme mechanisms, member reach and engagement, and barriers and enablers to participation.^19 20^ Quantitative process indicators and qualitative themes are presented narratively, with qualitative findings used to contextualise observed quantitative patterns.

The study team maintained records of all contact with liaisons (eg, phone conversation summaries and email logs), documented field visit observations and kept a log of education and training sessions, including attendance numbers. During screening or at the point of withdrawal, organisation liaisons provided reasons for their decisions to engage or not engage. These reasons were categorised into broad themes created through an iterative process, with the study team discussing and refining categories as new reasons emerged. Web analytics from the platform (REDCap) used for intervention dissemination included metrics on engagement with the intervention material sent via email, such as whether they were opened and viewed.

At the 12-month follow-up, participants in intervention clusters were invited to provide feedback on the intervention and its implementation via surveys and focus groups. Respondents were voluntary or opt-in, and survey items asked participants to reflect on their recollection of CPR training at their organisation over the preceding 12 months (see online supplemental S2).

Organisation liaisons were interviewed online using the secure cloud-based video conferencing service Zoom to obtain feedback on programme facilitation and understand factors related to intervention delivery and member engagement. A purposeful, maximum variation sampling strategy was used for liaison interviews to capture a range of perspectives across key characteristics. Liaisons were approached to maximise variation across organisation types (eg, social, multicultural, faith-based groups, sports) and locations (metro and regional) and where feasible, by key demographic characteristics (gender, age group). Liaison recruitment was iterative with invitations directed towards under-represented organisation types, locations and participation levels (including minimal or no member engagement) as feasible. The final mix of participants is summarised in online supplemental table S4.

For focus group discussions, participation was voluntary: participants indicated interest (opt-in) and consented to be contacted when invited to complete evaluation surveys and participate in the group discussions. Focus groups were scheduled based on participant availability and feasibility within recruitment constraints, resulting in three group discussions. All participants were invited to reflect on the design and delivery of the intervention components. The discussion guides and survey questions are listed in online supplemental 2.

### Data analysis

Categorical variables are presented as counts and proportions, and continuous variables are summarised as means with SD for approximately normally distributed variables and medians with minimum and maximum ranges or first (Q1) and third quartile (Q3) or IQR for skewed data. Where possible, data are described by subgroups of interest: organisation type (social/sport), location (urban or regional) and size of membership cohort (small/moderate to large).

Given the variability in intervention delivery and member engagement across intervention clusters, a pragmatic scoring approach was developed to summarise implementation using available indicators. Each cluster was assigned a score from 0 to 4 based on the extent of intervention delivery and member engagement (online supplemental table S1). A score of 0 was given to organisations that withdrew or had no engagement. A score of 4 reflected full delivery of all four components over 12 months and engagement of more than 20% or more than 30 members with at least one component. This score was developed pragmatically (delivery and uptake thresholds set by the study team) for descriptive comparisons and was not validated as a measure of implementation fidelity or intervention uptake.

Content analysis was used to summarise the study records and field notes collected throughout the study period.^21^ Interviews (30–45 min) and group discussions (1.5 hours) were audio-recorded, transcribed verbatim and independently coded by two researchers from the study team who subsequently reached consensus codes. When consensus was not reached, these were discussed with a senior researcher (CC/JR) until agreement was reached. Thematic analysis was used to summarise the patterns that emerged from the data collected (interviews, focus group discussions and field observations), employing both inductive and deductive logic.^22^ NVivo V.12 was used for coding and theme development.^23^ 10 committee liaisons participated in interviews, and 14 intervention cluster participants provided feedback across three focus group discussions (online supplemental table S4).

Findings and patterns emerging from the analysis of data from multiple sources were considered together during interpretation and are presented in alignment with the UK Medical Research Council (MRC) process evaluation framework.^15 16^ Interpretation was guided by the prespecified aims and MRC framework domains. The framework supports process evaluation across three areas: examining implementation (including delivery, structure, fidelity and reach), exploring mechanisms of impact (how the intervention works) and considering contextual factors influencing implementation.^24^

Reporting of this process evaluation follows the Standards for Reporting Implementation Studies (StaRI) guidelines^25^ and a completed StaRI checklist is provided as an additional file.

### Patient and public involvement

The public and members of the community were involved in the design of the participant evaluation survey items. The initial survey questions were pilot tested with members of the public who represented the target study population. Their suggestions and feedback were incorporated when finalising the survey questionnaire. Committee members at social and sports organisations participating in the FirstCPR study where the survey was administered were closely involved in facilitating the implementation of the intervention at their organisations.

## Results

Aligned with *Aim 1,* we begin by presenting organisational enrolment and determinants of study participation. We then address A*im 2* by reporting intervention implementation across intervention clusters, organised by reach, uptake and engagement. Quantitative data are presented alongside qualitative findings drawn from interviews, focus group discussions, researcher observations and field notes, and liaison communications. See tables1  2 and online supplemental tables for illustrative quotes.

**Table 1:** Enablers to organisation enrolment

Themes, subthemes and codes	Illustrative quotes
**Theme: motivated community champion (organisation liaison)**	
Liaison motivation and values Altruism/service oriented	“…I think it can happen to anybody, and I think this is a great program. I mean, honestly, if we can save one life…you have achieved a lot, yeah” – P1 “I have always been interested, put it this way, in helping my fellow man… so straightaway I was always interested” – P3
**Theme: organisation alignment with programme**	
Alignment with organisational values, purpose and community benefit CPR aligned with organisation mission/values Liaison had liberty to decide on study enrolment	“We said yes [to enrol] because it fits our mission. We want to help people learn how to care for each other effectively, and knowing CPR fits in that mission” – P7 “Our aim is to work in the interest of senior citizens, improve their quality of life, carry out cultural, educational, benevolent, social and recreational activities for their benefit” – Liaison at a culturally diverse seniors society, Urban location - Researcher notes
Member demographics increased perceived relevance	“With as many old people as we have, we need to be competent…need to be confident” – P3
Existing AED as a prompt to consider training AED usefulness increased perceived need for training	“…part of the reason that we did it…one of the youth groups bought the AED …and decided that they wouldn’t install it until they had people trained in it.” – P16 “…because we have a defib… that training is actually very useful…at least there’s a defib, and they know how to operate it to save someone’s life.” – P10
**Theme: trust and cultural considerations**	
Credibility of a university-led research initiative No-cost training reduced barriers Language support increased accessibility Bilingual staff enabled rapport building and reach to diverse community groups	“university link endorsement was good …it’s that quality endorsement, you know it’s legitimate rather than someone that’s going to try to spam you or scam you.” – P14 “… and we have to advertise for people, this is our three options of classes, free training and everything is provided” – P10 “…our group in general…if we deliver in English then the majority wouldn’t have understood, so we booked an interpreter…was quite good” – P20

(P) refers to interview/focus group participants - see online supplemental table S4 for participant characteristics. Quotes and subthemes when labelled as researcher field notes are drawn from communications recorded by the study team. Also see ‘Reasons for ineligibility and refusals’ in online supplemental section 4.

AED, automated external defibrillator; CPR, cardiopulmonary resuscitation.

**Table 2:** Contextual and intervention design factors influencing member interaction and engagement

**Theme: contextual/extraneous factors** (illustrative quotes)	
Timing and external disruptions (eg, COVID-19)	“COVID really disrupted it…it was spread over a long period… people… they couldn't be bothered with getting involved, you know, and that’s probably just a casualty of the pandemic…” – P3 “I think it might have just been timing… people were still concerned about going out … still a bit apprehensive” – P5
Competing priorities	“Not interested in participating anymore as we are in the off season” – Researcher Field notes; Football club, Urban location
Local champion/liaison enabled implementation	“You have to find somebody in an organisation that will grab it and work with it” – P4 “I’ve done research before…I know how hard it is to get participants, so if we could help… I was pleased to help” – P7 “…second barrier was just trying to find the time, as I was trying to build up the business consuming all my time” – P8
Organisation size	“The smaller clubs… [members] have big interest … whereas bigger the club gets…people just turn up go to dinner” – P2
Opportunities to gather	“Online communication is a compromise…we are relational beings. Being in person is the best way” – P7
Supportive committee	“…It all depends on the committee. If willing and they put the hard yards…. these things are definitely possible” – P1
Real incidents	“… after a participant of one of the FirstCPR sessions witnessed an arrest and … provided assistance …other members of this club have found out …requested for more training due to increased interest” – Research notes “… a young man collapsed when playing … has shaken the community…no one around knew any CPR …a very fresh event … likely to encourage members to take up training” – Researcher notes
**Theme: intervention design and delivery-related factors** (illustrative quotes)	
Campaign-style: novelty, paced messages	“…it was refreshing …after COVID, …it gave you another outlook… ‘we are going to get together’ … I’m waiting for my next bit to happen” ” P5 “…you do it in own time… wasn’t in your face …was in the background…we’d say ‘oh did you see the latest one” – P5
Incentivisation and fee-free training	“…maybe something as simple as lunch will be provided or something like that…especially if you are trying to take the best part of a day or part of a day out of a person’s weekend” – P8
Cultural tailoring	“Chinese trainer, material in Chinese… do require some English for some issues…listening to the instruction from the machine (AED)… needs some basic English… but having a Chinese trainer just makes them more comfortable” – P6 “… have two instructors, one for-…one female …and one male …people might not feel comfortable talking to the opposite genders” – P10
Recruitment method	“We have you know about 8500 members, and we probably only e-mail to about 3500 of them” – P2 “…had people not had inboxes full, they probably would be more inclined to… do it” – P17 ‘She refuses to send the survey link …insists that no one will fill it - “come and show us how it’s done… help us if we get stuck in completing the survey” – Researcher field notes
Logistics and scheduling constraints	“…we were trying to put it on Facebook…our media director*…*did not come to the party quickly …so a delay,” – P1 “*…*have a class on the weekend… late night…school holiday…need to accommodate according to their timing” – P10 “… just afternoon sport, *…* premier type players coming in and going out …was just a very busy time.” – P14
Awareness-building as a prerequisite for engagement and uptake	“… before sending the baseline survey to their members, they would like to have a Zoom meeting … explaining the program to their members. … promotion activities*…*” – Researcher notes, large multicultural faith-based organisation “spreading awareness … cascading effect…motivate, they actually will bring other members” – P9
Trust via university plus organisation endorsement	“the university link endorsement…quality endorsement, you know it’s legitimate.” – P14 “Club endorsement number one because it’s not just, you know, someone saying…” – P14
Hard to engage those who train regularly in First aid/CPR	“…some people have done that course ten, twenty times in their life…sick of listening to the same thing again, again…they just wanted to do the practical and refresh themselves.” – P10 ‘*…*Tried engaging with players before/after they had played …many had first aid training through their employment’ – Researcher field notes
Positive spillover beyond the organisation	“made people that were participating… go back to the other clubs and talk about what they were learning….one of the clubs has now bought a defibrillator. So, it’s actually spreading …it spread to other clubs” – P4 “*…* have shared your FirstCPR with few of my friends, I always tell them, share it with others as well” – P13
Preferences and suggestions for the digital component Brief, simplified learning valued Reinforcement, spaced learning Broadens reach and reduces cost Content sequencing	“…very simplified …refresher courses …we spent hours doing that, here *…*same thing done in minutes…very clear” – P13 “educational, informative …the subsequent ones a good refresher… good space in between” – P18 “Some of the things are repeated, that’s OK, in the sense for us to reinforce” – P13 “It reduces cost, broadens access to people and then hopefully more people will actually do CPR” – P7 “seemed to be out of sequence*…* you seem to be progressing… then back to going over again what you would do” – P3
Diminished engagement over time	“I would receive an email… look, I've seen this before. I’ve had enough I don’t really feel the need to watch anymore…law of diminishing returns kind of kicked in…the earliest communication, make those the best” – P7 “…condense it*…*do it over 2–3 months maximum…then maybe a follow-up course*…*12-18 months down the track” – P3 “…that session alone satisfied our congregation *…* after that, lost a bit of urgency …people were satisfied” – P7
In-person preference	“How important is accreditation? … As opposed to…getting familiar with…with the process on a manikin*…*” – P18
Real-life narratives	“*…*people are driven by how they feel*…*until people hearts are engaged…other noise …will take their attention” – P7
Trainer quality, practical tips	“…working in teams …really like the collaboration aspect…someone calls for help and then swap over and stuff.” – P14

(P) refers to interview/focus group participants; see online supplemental table S4 for participant characteristics.

AED, automated external defibrillator; BLS, basic life support; CPR, cardiopulmonary resuscitation.

### Organisation enrolment and factors influencing participation

#### Organisation screening, eligibility and enrolment

**Figure 2:**
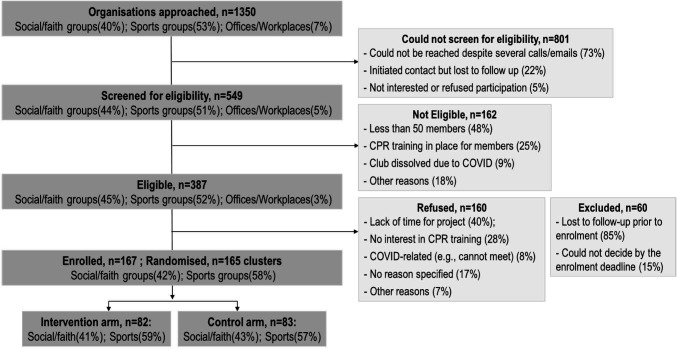
Organisation screening and recruitment. CPR, cardiopulmonary resuscitation.

A substantial proportion (n=801, 59.3%) of a large pool of organisations (n=1350) could not be screened for eligibility, mostly due to difficulties with contact and non-responsiveness (figure 2). Of the 549 organisations screened, n=62 (29.5%) were ineligible based on study enrolment criteria (eg, membership size, physical location, existing CPR training). Of the 387 eligible organisations, n=160 (41.3%) declined to participate and n=60 (15.5%) were excluded as lost to follow-up (could not be recontacted to complete enrolment) or were unable to make a decision on participation prior to enrolment deadline. Eventually, 167 (43.2% of eligible) sports, social and faith-based organisations were enrolled, most n=141 (84.4%) were in metropolitan areas (online supplemental figure S2). We were unable to recruit any offices/workplaces.

#### Barriers to organisation enrolment/participation

The key reasons for refusal or inability to participate were a lack of time to support the facilitation of the trial activities or a lack of interest in bringing CPR training to their cohort (figure 2). The COVID-19 pandemic also impacted participation, cited due to uncertainties about the safety and feasibility of member gatherings. Enrolment was also challenging in organisations when a larger committee had to decide whether to participate. A list of other reasons for ineligibility and refusal to enrol is detailed in the online supplemental tables S2,S3.

#### Enablers to organisation enrolment/participation

##### The community champion/organisation liaison

Participation was heavily dependent on the contact person, that is, liaison in the organisation (eg, presidents, secretaries, events committee members), who decided on study participation. In particular, their time and motivation to bring such activities to their members. Their interest in disseminating our training to their membership was heightened by awareness that ‘(a cardiac arrest) can happen to anybody’. Liaisons with a background in health or research were also more likely to value and support the programme. Memory of a recent cardiac arrest incident was noted as a motivator to enrol (table 1).

##### Organisations’ alignment with programme

Organisations with a strong community focus or a history of supporting health-related activities were more receptive to the intervention. This included church groups and other faith-based organisations that placed importance on learning lifesaving skills as a contribution to the community. Additionally, organisations serving older populations showed a strong interest in the training due to their perceived higher risk of cardiac events. Having an AED installed at the organisation prompted the leadership to consider enrolling, as they were often aware that most members did not know how to use it.

##### Trust and cultural considerations

The involvement of bilingual study team members played a key role in building rapport and encouraging participation from organisations with a culturally diverse population. Offering intervention materials in participants’ preferred languages further enhanced reach, particularly among groups where English was not the primary language spoken. Enrolment was also facilitated by the provision of free training, and the credibility associated with a university-led research initiative, which helped build trust in the programme and providers.

### Intervention implementation

#### Reach

Reaching out to members to invite them to participate in the intervention was a collaborative effort between the organisation liaisons and the study team (figure 1). The theoretical reach across the 82 intervention clusters was approximately 35 531 registered members. However, it was not possible to determine how many members were aware of the study or were invited to access the intervention material or attend FirstCPR sessions. By 12 months, eight (15.6 %) intervention organisations withdrew, six of these were sports groups (online supplemental table S5). Reasons for withdrawals included limited committee capacity/time, competing operational priorities or disruptions (eg, flood damage, COVID-19-related restrictions, off-season), as well as perceived limited benefit or low member interest (online supplemental table S5). A greater number of control clusters withdrew (n=5, 18.1%), and an additional 28 (33.7%) were lost to follow-up. Common reasons for withdrawal were similar to those of the intervention group but also included a change in leadership with reduced interest. Reasons for loss to follow-up were largely unknown as organisations did not respond to contact attempts.

#### Uptake

##### Digitally delivered messages

The link to sign up for the digitally delivered educational messages (email/texts) was distributed to liaisons at all 82 intervention clusters; however, only 72 (87.8%) circulated it to their members. A total of 987 members across 69 organisations opted in. The proportion participating in this component did not differ by membership size (<200 members: median 5%, range 0.5–35%; ≥200 members: median 5%, range 0.2–13%). Message delivery by email (77%), with only 14% opting for text messages and opting for both email and texts. Language preferences were English (91%) and simplified Chinese (9%).

##### In-person sessions participation counts

The 1-hour group session was delivered at 32 organisations to a total of 742 members with a median of 14 people per session (Q1 7, Q3 25). Minor adaptations were made to meet organisational needs (eg, online sessions, provision of interpreters). The 3-hour accredited training session was held at 22 organisations and attended by 406 members (median 13 people per session; Q1 8, Q3 24).

### Engagement

It was not possible to track text viewership, but about a third (33.3%) of the email recipients viewed at least one message. Social media tiles, newsletter snippets, posters and brochures were delivered to liaisons at 78 (95.1%) intervention organisations for display or distribution, but their distribution to members and their engagement with the material could not be accurately assessed.

**Figure 3:**
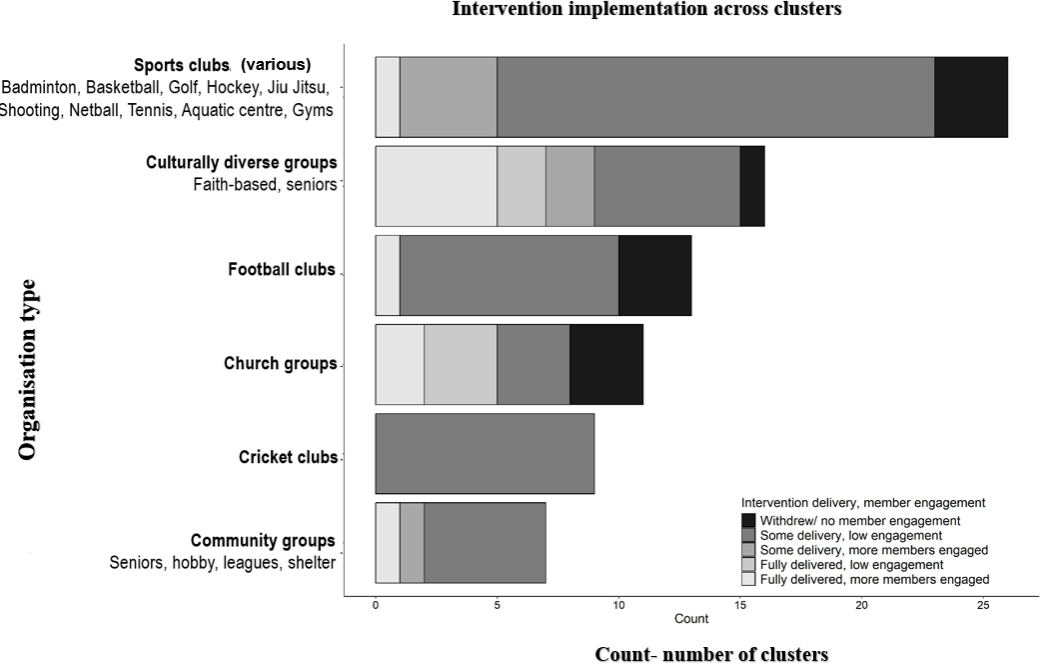
Variable delivery of intervention components and member engagement.

The majority (78%) received some form of the intervention materials, with 74 organisations engaged with at least one activity and 15 in all activities. Participation rates among members showed great variability in engagement levels across organisations, including within the same organisation type (figure 3). Integrated findings from researchers’ field notes and interviews with liaisons suggest that high participation occurred when multiple enabling conditions were present.

Field visits played a crucial role in facilitating interaction with members and boosting engagement. These visits were facilitated through organisational liaisons. Visits were conducted at 87% (13/15) of the most-engaged organisations (all components fully delivered) but only 27% (18/67) of the less-engaged groups. We identified ‘most’ and ‘least-engaged’ organisations based on our internal scoring system (figure 3, online supplemental table S1, figure S3).

The scoring system we developed to summarise intervention implementation and member engagement showed that members were less likely to engage with all components of the intervention. Relatively greater uptake and engagement were observed at some of the faith-based groups and organisations with culturally diverse populations compared with sports groups (figure 3). However, when key enablers were lacking or underdeveloped, engagement was minimal - irrespective of group type (online supplemental figure S3).

### Participant feedback on intervention (acceptability and suggestions)

Participant feedback at 12 month follow-up provided additional insight into the acceptability and perceived usefulness of the intervention components. In total, 189 participants from 46 intervention clusters (51% females, median age 62 years, 55% Australian-born) provided free-text feedback on intervention activities. Overall, participants found the education and training was informative, easy to follow and beneficial in learning life-saving skills. They liked the delivery of small learning segments to help reinforce key concepts and the ease of access via text message and emails. Messaging and materials were reported as simple and easy to understand (eg, simplified ‘Call-Push-Shock’ message). Participants valued the opportunity to practise skills and noted that discussion of real-life examples and personal stories made the information more engaging and made the training more impactful. AED locations and usage guidance were noted to be valuable learning.

Suggestions to improve the intervention included making the content more interactive, including quizzes and real-life scenario-based training. Some requested more detailed information, ongoing brief training and integration with full first-aid training. There was mixed feedback on the mode of training, with some preferring apps, videos, digital media and others preferring in-person training. Several improvements to the training were suggested, including shorter sessions, better seating arrangements and guidance on performing CPR for individuals with mobility challenges. Participants emphasised the importance of an inclusive approach, ensuring CPR training is accessible regardless of language or certification requirements (online supplemental table S6). Common questions asked by participants in in-person sessions are listed in online supplemental section 5 (box 1).

### Factors influencing intervention implementation and member engagement

Factors influencing implementation and engagement are grouped into two main themes aligned with the UK MRC framework: (1) contextual/extraneous factors and (2) intervention design/delivery factors. Subthemes are noted below and illustrative quotes are in table 2.

#### Contextual/extraneous factors

##### Timing and external disruptions (eg, COVID-19)

COVID-19-related restrictions in the study areas limited group activities in the first 8 months, delaying implementation and reducing willingness to congregate. Even as restrictions gradually eased, lingering concerns continued to limit participation, potentially exacerbating existing barriers to enrolment. However, in some organisations, as restrictions eased, our programme was a welcome activity to regroup, and digital components helped maintain engagement among members who were unable to attend in person.

Organisations in some of the regional areas were also affected by extreme weather events, including floods and bushfires. Several text message scams occurred during the same period, and a few participants expressed hesitancy to open links in texts (field notes). Unfortunately, we were unable to quantify how much these events affected engagement with the intervention.

##### Competing organisational priorities

Liaisons cited competing organisational priorities such as ongoing community initiatives or seasonal sports schedules as influencing participation levels. Field notes and liaison communications indicated that seasonality constrained engagement in sports clubs, particularly during the off-season (table 2).

##### Local champion/liaison enabled implementation

Interviews and field/researcher notes consistently highlighted the community liaison as a critical enabler and facilitator of implementation. Their commitment and time availability influenced the extent of delivery and member participation. Altruistic, service-oriented attitudes were commonplace among those who facilitated the programme. Liaisons who could promote and normalise the training sessions within existing social structures were more successful at engaging members. However, at some organisations, even highly motivated and engaged liaisons expressed surprise and frustration when member interest remained low despite multiple efforts. At sports organisations, liaisons were frequently time-poor balancing full-time employment with volunteering club duties that constrained support for new activities despite substantial study team support (eg, provision of advertising materials, organisation of in-person sessions).

##### Organisation size and engagement dynamics

Smaller clubs displayed a more hands-on approach to participation. Larger organisations sometimes faced challenges securing engagement from a dispersed membership base.

##### Opportunities for members to gather

Engagement was notably stronger within social and faith-based groups. These were organisations where members could gather. Members of sporting organisations were mostly constrained to meeting specifically for training and games, which limited opportunities to engage in non-sporting activities, and it proved challenging to get members to stay back after their sport. Furthermore, sports grounds were not as conducive to engaging with members.

##### Supportive organisation committee

A key enabler to intervention implementation success was the presence of a supportive leadership team or committee, which facilitated engagement and integration of the programme into existing organisational activities.

##### Real-life incidents

Stories from participants about providing bystander assistance during cardiac arrest incidents following their FirstCPR training generated increased interest among other members in joining the intervention activities. Another catalyst at a sports organisation was the account of a life saved by the availability of an AED. An incident involving the unfortunate loss of a young community member to cardiac arrest triggered members of the community to focus on engaging in the FirstCPR training.

### Intervention design and delivery-related factors

This domain explores how specific features of the intervention design, its delivery and its interaction with the various components may have influenced participant engagement.

#### Campaign-style: novelty and paced messages

Integrated qualitative findings indicated that participants valued having both digital and in-person components, citing learning flexibility and reduced barriers such as cost and time. Participants described messages sent (every 2–3 weeks) as useful reminders, and several noted that staggered delivery supported self-paced engagement and promoted informal discussions with others. Some described the shared delivery schedule as creating a sense of shared experience within the group. Brief, practical in-person sessions with opportunities to practise skills and AED use were commonly preferred, while accredited training was mainly valued when it was required for work.

#### Incentivisation and fee-free training

An important factor influencing participation was the fee-free nature of training. Additionally, participants suggested including strategies such as hosting training alongside social events to further enhance participation. In line with this, one strategy involved aligning the informal educational session with a cultural celebration, with the intent of capitalising on the large number of members already gathered for the occasion, to increase visibility and attendance.

#### Cultural tailoring

Participants and liaisons appreciated the customisation of intervention material and training delivery in the language of preference, and some emphasised the need to accommodate cultural sensitivities, for example, preference for separate sessions for male and female groups. Participants noted the need to be aware of key English words likely to be needed in an emergency.

#### Recruitment method

The programme relied on liaisons to distribute study links to organisation members (via bulk email or via newsletter with sign-up links). This approach created several barriers. The connection to the research requirements of consent and baseline survey participation to access the digital intervention component deterred participation. Digital links posed challenges for organisations with elderly members. The indirect and passive recruitment method made it difficult to determine actual member reach or engagement levels.

#### Logistics and scheduling constraints

Delivery of educational materials was facilitated via liaisons at participating organisations that sometimes led to a delay in the delivery of study links to members, and at other times, there were logistical challenges and scheduling constraints (for in-person sessions), particularly at sports groups where members congregated only for training or games.

#### Awareness-building as a prerequisite for engagement and uptake

Participation in the study activities was often proportional to the level of awareness generated about the importance of learning BLS skills. When the research team met with organisation members to explain the study, participation and engagement rates were higher. Word of mouth among members was another way that encouraged participation.

#### Trust via university plus organisation committee endorsement

There was trust in the educational programme delivered by university researchers. Furthermore, the organisation liaisons’ vetting of the study was also an important trust factor for members.

#### Hard to engage those who train regularly in First aid/BLS

FirstCPR’s format, including intervention length, duration, frequency and research participation requirements may not have appealed to sporting club members who train regularly in First Aid or BLS. Recruitment challenges, such as at one of the sports organisations, were noted as a lack of interest because most of their members were health professionals who already received regular training. Participants from other sporting organisations suggested that shorter one-off sessions focused on brief practice and learning refreshers might be more appealing to this audience.

#### Positive spillover beyond the organisation

Instances of spillover effects extended beyond participating organisations were noted. Participants shared FirstCPR messages with friends and across club networks. In one instance, discussions about the programme with another club in the town prompted that club to purchase a defibrillator.

#### Preferences and suggestions for digital components

Participants described the messages as simple, succinct and clear, allowing essential content to be covered in minutes rather than hours. Regularly spaced messages were viewed as helpful refreshers that reinforced key steps without feeling overwhelming, as they were spaced every 2–3 weeks. Digitally delivered materials were seen to reduce cost and broaden reach to members who could not attend in person, supporting wider community access. Some participants perceived the messaging to be out of sequence, noting that advanced scenarios sometimes appeared before foundational concepts were fully covered. One participant observed that as understanding of CPR progressed, the content reverted to earlier stages, creating a sense of moving backward rather than building on prior knowledge. Participants recommended a progressive build and suggested making the material more engaging and less repetitive (eg, multiple-choice questions to test knowledge, videos that encourage at-home practice using props such as pillows).

#### Diminished engagement over time

Opt-in text messages every 2–3 weeks attracted early interest but engagement attenuated over time with fewer messages opened. This decline may reflect waning interest in receiving varied content on the same core topic, even though some participants valued the refreshers. Some participants reported that engaging with a single component, for example, in-person sessions met their learning needs and led to reduced interest in accessing other components afterwards.

#### In-person preference

The 45-minute to 60-minute educational session combined informational content delivered via pictorial slides with interactive discussion covering cardiac arrest recognition, response steps, common concerns and skills demonstration. Attendees were encouraged to ask questions and invited to practise CPR and learn to use an AED. Although unaccredited, this session was popular among the elderly for whom accreditation was not a priority.

#### Real-life narratives and lived experiences

A majority of members enjoyed the brief, interactive and informal in-person sessions where opportunities to interact and ask questions were encouraged. It incorporated elements of storytelling and real-life narratives often delivered by engaging, experienced trainers.

#### Trainer quality and practical tips valued

Among members who attended the formal 3-hour accredited sessions, there was gratitude and appreciation for the opportunity to learn, practise skills and receive practical tips from experienced trainers.

## Discussion

This process evaluation of the FirstCPR programme provides real-world insight into factors influencing participation and engagement with community-delivered BLS education and training opportunities. Few studies have examined the implementation of community-based BLS/CPR training interventions.^26^ This theory-informed multimethod evaluation adds detail on factors influencing reach, uptake, engagement and delivery within community organisations. Guided by the UK MRC framework for complex interventions, these findings can inform the design, scale-up and adaptation of similar programmes.

Delivering BLS interventions via social organisations and networks enabled reach to a broad range of groups, including those less likely to access training through traditional models. However, generating strong interest in both intervention engagement and survey participation proved challenging. This may reflect that BLS public health initiatives differ from other health promotion and education programmes targeting existing health risks or conditions, as the benefits may be perceived as less immediately relevant. Engagement levels were lower than anticipated and varied considerably across organisations, with increased uptake at social and faith-based groups. A complex array of factors influenced participation and engagement, reflecting the influence of intervention design and delivery format, organisational features, liaison attributes and other contextual factors.

### Organisational factors and the critical role of the community liaison/champion

The notable success with some social and faith-based groups likely reflects alignment between the intervention and organisational values, regular opportunities for members to gather for religious or social gatherings, and the pivotal role of organisation liaisons. Similar findings have been reported in BLS training initiatives in underserved communities, where churches and local champions have been key enablers that drove intervention uptake.^27^ Partnerships and the intersection between public health and faith-based groups have previously been noted in the literature, highlighting the benefits of congregational-based health promotion, especially in reaching diverse groups and underserved communities.^28^,^30^ A qualitative study involving interviews with public health and congregational leaders in North America reported on the importance of congregational capacity and trust as key elements of establishing partnerships and collaboration successfully.^30^

The role of community leaders in enhancing engagement with health promotion interventions has been emphasised previously,^31^,^33^ and our study reinforces that established trust and relationships can enhance programme reach.^34^ These liaisons served as a gateway to both organisational participation and member engagement, and successful implementation was linked with their motivation and ability to support programme activities. They are often long-standing members of the community and usually have a good understanding of their community’s needs, enabling better tailoring of intervention delivery to address their members’ preferences.^35 36^ While the intervention components were largely delivered as intended, variations in engagement reflected differences in liaison involvement and member interests. These findings are likely to be transferable across a range of social organisations and clubs worldwide, though specific formats and delivery modes may vary by context, and barriers and facilitators to implementation must be recognised and addressed through early engagement with local champions.^37^

Organisations reporting recent cardiac arrest incidents were also more likely to prioritise CPR training, potentially reflecting heightened perceived risk. Risk perception that OHCA can occur close to home may foster a more positive attitude toward learning/refreshing BLS skills.^38^ Similar to our observations, survivor narratives and fact-based content highlighting real-world risk contexts (eg, the majority of arrests occur in homes) have been identified as effective knowledge translation strategies and can increase public risk perception and improve engagement with training.^38^,^40^ Participation increased in some groups when the study team had the opportunity to explain the importance of CPR training and address questions through interactive awareness-raising activities.

### Challenges in community engagement

Despite the enablers identified, member uptake and engagement were lower than anticipated. Tying CPR training to research may have been off-putting to some individuals, echoing findings that participation in community interventions can be lower when coupled with additional research burden or survey participation.^41^ Additionally, participating in CPR education and training may not elicit the same level of prioritisation as participation related to an existing health condition.^38^ Non-participation may also reflect prior CPR training among some groups, for example, through the workplace or sport requirements. Future efforts could consider alternative ways to assess impacts, such as administrative data or employing comprehensive community-based participatory approaches early on to facilitate participation and support engagement.^42 43^

Challenges in gaining committee approval included the view that training a select group of leaders (eg, volunteers or coaches) was sufficient, reflecting a limited understanding of the importance of broader community reach in CPR training. While the explanation of the study’s wider aims occasionally improved engagement, this was not consistent.

### Learning preferences

There was limited interest in accreditation among elderly participants who instead valued the brief interactive sessions with opportunities to practise skills and AED use. This supports prior evidence that elderly community members prioritise skill confidence over certification^44^ and has implications for framing public health messaging. Feedback on digital snippets emphasised the importance of logical content sequencing in digital learning.^45^ Maintaining progression may enhance learning motivation, consistent with broader digital health education literature. The disengagement we observed after initial messages suggests that digital reinforcement should be less frequent for CPR training but more novel and targeted to reinvigorate interest.

### Culturally responsive approaches and flexible delivery

Culturally responsive and tailored approaches, including a diverse study team of bilingual staff, translated material and interpreters, were valued and improved participation.^46 47^ Additionally, hosting sessions at the organisation’s own venues also supported participation. These strategies align with equity-focused approaches to tailoring interventions and are likely transferable to multicultural contexts internationally, though specific adaptations will vary by local context.

### Limitations

Given the community setting and the nature of the intervention design, we encountered practical constraints in collecting every piece of data on study-related activities and in accurately quantifying engagement. For example, engagement with educational messages delivered through organisations’ social media channels or newsletters could not be quantified. Comprehensive process measurement can be resource-intensive and not always feasible in real-world community settings.^48^ Recruitment and implementation occurred during the COVID-19 pandemic period, which likely exacerbated barriers to participation. Online components helped to maintain some continuity of access.

There is also a risk of selection bias and limited generalisability as only 167 of 387 eligible organisations participated. Those who took part may have had greater capacity, motivation or leadership support for BLS training than those who declined or were not retained, limiting transferability of findings to time-poor organisations or those with limited facilitation capacity. Attrition over follow-up, with additional dropouts before the 12-month assessment, may also have influenced observed implementation patterns. In addition, there was limited input from non-participating organisations and from members who did not engage, which may have introduced bias, missing perspectives and an incomplete understanding of barriers to participation. Focus group involvement was opt-in and self-selected, further limiting the representativeness of views. Similarly, 12-month feedback was collected through voluntary surveys, which could introduce selection bias, for example, if respondents were more engaged with the intervention than non-respondents. As survey items required participants to recall activities conducted over the preceding year, responses may also be affected by recall bias. As a result, reported per population and fully represent the broader intervention population, and estimates of acceptability or usefulness should be interpreted with caution.

The implementation scoring system used to summarise delivery and engagement across clusters was developed for this study and has not been formally validated. Thresholds were set pragmatically by the study team and may have introduced misclassification (eg, organisations with different engagement patterns receiving similar scores). The score should therefore be interpreted as a descriptive summary of implementation variability, rather than a validated measure of fidelity or uptake.

## Conclusion

A comprehensive process evaluation provides insight into the implementation of a multimodal community BLS intervention and highlights determinants of participation and engagement in real-world settings. Participation was enabled by early organisational leadership support and, most importantly, a motivated liaison/champion, while time constraints and competing priorities reduced enrolment and ongoing participation. Reach and engagement varied widely across organisations; uptake was stronger in social and faith-based groups where members regularly congregated, and facilitation was actively supported. Participants preferred brief, simplified learning modules, opportunities to practise skills and culturally tailored delivery, which improved accessibility. Minimising research burden and supporting liaisons with feasible delivery options (brief, practical, flexible and culturally responsive formats) may strengthen engagement and improve scalability of community-based CPR training. Shifting from traditional classroom-based CPR training to flexible, outreach approaches with opportunities for deliberate practice may expand reach and access and strengthen community preparedness for OHCA emergencies.

## Supplementary material

10.1136/bmjopen-2025-113343 online supplemental file 1

## Data Availability

Data are available upon reasonable request.
