# Supplementary material for: Process evaluation of a basic life support educational intervention (FirstCPR cluster randomised study) delivered at community organisations in New South Wales, Australia
Source: BMJ Open. 2026 Apr 7;16(4):e113343. doi: 10.1136/bmjopen-2025-113343 (PMC13064210; doi:10.1136/bmjopen-2025-113343)
Supplement: online supplemental file 1 [file bmjopen-16-4-s001.pdf]

Process evaluation of the FirstCPR study_ Supplementary information

Table of Contents

1. FirstCPR intervention and activities related to implementation 2
Figure S1: FirstCPR intervention components 2
2. Data collection instruments 3
1. Survey feedback – intervention cluster members 3
2. Discussion guide: Interviews with committee liaisons at participating intervention organisations 5
3. Discussion guide: Focus group discussions with intervention arm participants 8
3. Nominative labelling to summarise intervention implementation 11
Table S1: Nominal categories 0 to 4 summarised intervention delivery to organisation and member engagement 11
4. Organisation recruitment 12
Figure S2: Screening and recruitment by organisation location (Urban / Regional area) 12
Table S2: Reasons for organisation ineligibility 13
Table S3: Reasons for organisation refusals 15
5. Intervention implementation at intervention clusters 17
Figure S3: Summarising intervention delivery and uptake by organisation type using the scoring system 17
Table S4: Features of committee liaisons (interviewees) and focus group discussion participants 18
Table S5: Features of intervention organisations that withdrew from FirstCPR 19
Table S6: Participant recommendations and suggestions for future community-based training 21
Box 1. Questions and comments by members in the audience at the interactive information sessions 22

1. FirstCPR intervention and activities related to implementation

Figure S1: FirstCPR intervention components

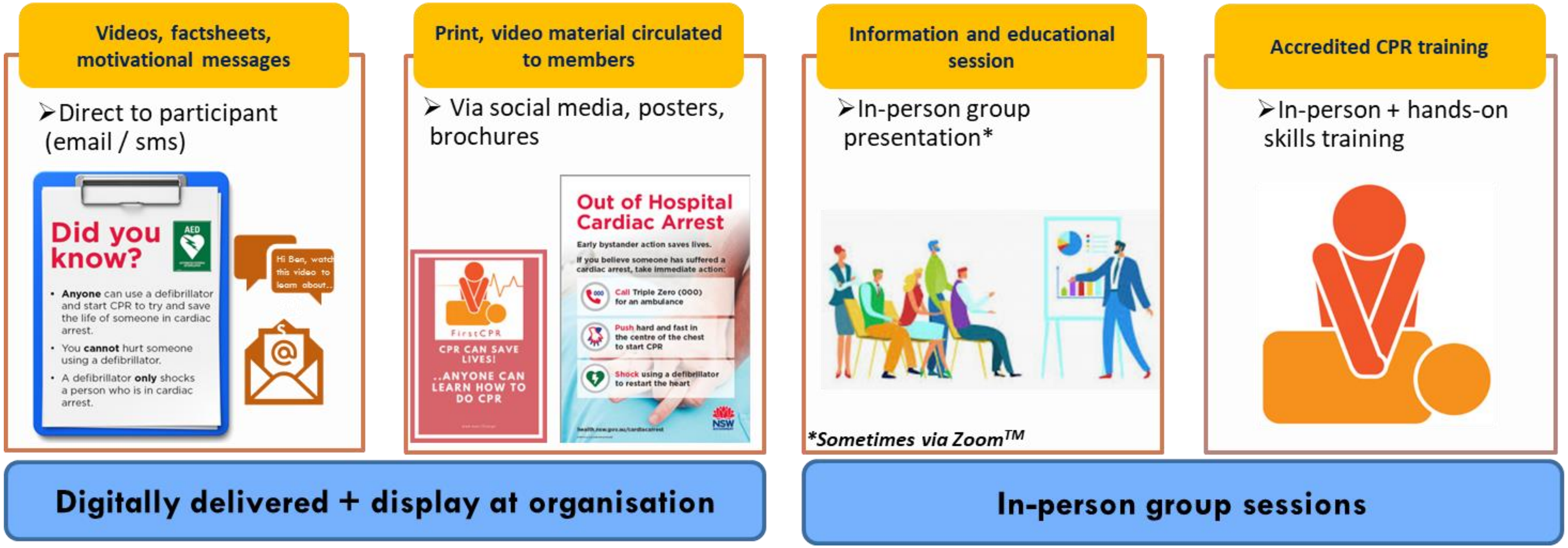

Extracted from Munot et al, 2025. FirstCPR: A pragmatic community organisation-based cluster randomised trial to increase community training and preparedness to respond to out-of-hospital cardiac arrest. Resus plus 2025 Vol. 23 Pages 100949. DOI: https://doi.org/10.1016/j.resplu.2025.100949. Reproduced here under the terms of the [Creative Commons Attribution License (CC BY)](#).

2. Data collection instruments

1. Survey feedback – intervention cluster members

Survey items excerpt from 12m evaluation surveys to seek feedback on intervention components<br><br>***Participants who completed baseline surveys and signed up to receive intervention messages delivered digitally via email/text (Cohort A) were asked:***<br>Please select whether you viewed or attended any of the following FirstCPR training interventions organised via your community organisation or club, in the last 12 months		
	Yes	No
1. Educational and informative videos and facts via text or email on how to respond to cardiac arrest	☐	☐
2. In-person or virtual group information session on how to respond to cardiac arrest (held by the Michael Hughes Foundation/Heart of the Nation)	☐	☐
3. Formal training in CPR and responding to a cardiac arrest (held by Surf Life Saving NSW)	☐	☐
(If yes to 1):<br>Can you comment on the digital content (videos and fact sheets) that you accessed and suggest how we can improve the training material?	……………………………………………<br>……………………………………………	
(If yes to 2):<br>Can you comment on the face-to-face group information session that you attended and suggest how we can improve the information delivered?	……………………………………………<br>……………………………………………	
(If yes to 3):<br>Can you comment on the face-to-face group information session that you attended and suggest how we can improve the information delivered?	……………………………………………<br>……………………………………………	

Do you recollect seeing any information on how to respond to a cardiac arrest being relayed on organisation social media pages or on posters/LCD screens at your club/organisation in the last 12 months?	□ Yes<br>□ No
Can you briefly comment on what you recall seeing and whether you found it useful?	……………………………………<br>……………………………………
***Participants who were members of the intervention organisations but had not signed up at baseline to receive digital messages (Cohort B) were asked fewer questions*:**	
Do you recollect seeing any information on how to respond to a cardiac arrest being relayed on organisation social media pages or on posters/LCD screens at your club/organisation in the last 12 months?	□ Yes<br>□ No
**If 'yes',** can you briefly comment on what you recall seeing and whether you found it useful?	……………………………………<br>……………………………………
Did you attend a CPR information session at your club or organisation in the last 12 months?	☐ Yes<br>☐ No
**If yes,** can you comment on the information session that you attended and suggest how we can improve the information delivered?	……………………………………<br>……………………………………

2. Discussion guide: Interviews with committee liaisons at participating intervention organisations

Note: One-on-one interviews online/phone/in-person; Proposed duration: 20-30 mins

1. Interview with committee liaison who was able to facilitate FirstCPR at their organisation

Objectives/Purpose of this interview:

- To gain an understanding of the purpose of FirstCPR program as perceived by CM, and to understand reasons for participation
- To understand the extent to which the program met the expectations of the organisation
- To identify barriers to successful implementation of program (as planned) or barriers to specific components of the intervention
- To identify factors/enablers that supported the successful implementation of the program or specific components of the program

Questions:

Before we begin- do you have any questions or general comments about the FirstCPR project?

- In your view – what is the purpose of the FirstCPR program? *(Alternatives/Probes: why do you think this study is being conducted?)*
- Why did you participate?
- What were your expectations of the program for your members?
- Were these expectations met?
- What components of the program did you think were most useful for your members? (if needed probe: Why do you say that)
- What components were less useful? (if needed probe: Why do you say that)

(Probes: components = digital (sms/email messages); in-person sessions- hour long session conducted by MHF, 2.5hour session by SLS, social media snippets)

[The next few questions discuss barriers and enablers of FirstCPR implementation. These questions will need to be tailored for each organisation after reviewing which components were implemented well / or not so well at the organisation- then ask questions as relevant]

- What did you like about this program?
- And what did you not like about the program?
- How could we have implemented the FirstCPR program more efficiently at your organisation?
- What were some of the challenges in facilitating the FirstCPR program? (If not discussed – explore whether they think COVID had an impact – and if so in what way?)
- Did you have any difficulties in accessing the components of the program?
- How much time did you spend and how many people did you need to facilitate the FirstCPR program?
- Would you participate in a similar program again – if not – why not?
- Have you implemented a similar program (e.g., CPR training or other education program) and can you share your experience with us? (Probe: Is there anything from those programs that could help improve FirstCPR?)
- Would you participate in other programs in the future?

- What factors within your organisation allowed you to carry out FirstCPR as planned?
- What key things would you suggest we should consider before carrying out similar programs in the future?

Close: Are there any other comments you would like to make before we end?

2. Interview with committee members at who were invited to participate but *chose not to* enrol for various reasons (~15-20mins)

Objectives/Purpose of this interview

- To gain an in-depth understanding of the reason for non-participation in FirstCPR
- To identify elements of a successful public health/education program at their organisation (if applicable and as per committee members' experience)

Before we begin- do you have any questions or general comments about the FirstCPR project?

To gain an in-depth understanding of the reason for non-participation in FirstCPR

- In your view – what was the purpose of the FirstCPR study?
- Can you discuss why you were unable to participate?
- What would enable your organisation to participate in similar programs in the future?

(Or What would have made our program more attractive to your organisation?)

To identify elements of a successful public health/education program at their organisation (if applicable and as per committee members' experience)

- Do you recall a similar public health/education program or campaign (e.g., CPR training, COVID vaccination drive, or other health education campaign) that was held at your organisation?

  - (If yes), can you describe the factors that enabled successful implementation of that program?
  - Can you describe any barriers to the implementation of that program?

CLOSE: Are there any other comments you would like to make before we end?

This discussion will be summarised in a research report. No identifying information will be used to report any finding. Thank them for their time and close.

3. Discussion guide: Focus group discussions with intervention arm participants

Note: Online via zoom: (~6-8 per group); Proposed duration: 60- 75 mins

Objectives / Purpose of this discussion (RA note: do not read aloud- in blue):

- To understand acceptability of FirstCPR among participants
- To explore reasons for participation
- To explore what they perceive could be reasons for non-participation in their communities / among friends/family members
- to understand the perceived effectiveness of the program
- to explore perceived effectiveness related with each program component or related with module of transmission (in-person / digital messages to individual via email/text / digital messaging to group via social media)
- to note suggestions for improvement by participants
- to discuss intended behavior changes among participants
- to discuss approaches and strategies to conduct similar education programs/campaigns among members of their communities

Questions:

- What did you think about the FirstCPR program? (to get initial reaction, without prompts)
- What do you think is the main purpose of FirstCPR?

What prompted you to participate in FirstCPR?

Why do you think other members of your organisation or family, or friends chose *not to participate* in FirstCPR? (Probe: If not mentioned – Do you think COVID impacted participation in this program ?)

In what ways did you participate in FirstCPR? (or What training / education materials did you access as part of FirstCPR over the last 12 months? If no response, prompt to remind them of components)

- a) Did you view the digital messages that we sent (via email/sms)?
- b) Did you attend any of the in-person education/training sessions
    - If yes which one: MHF or SLS?

- c) Do you recall seeing any social media message / group message / video related with 'responding to cardiac arrest' circulated on your organisation's Facebook page / WhatsApp group / newsletter / or similar platform that was posted by your organisation committee member/s?

Related probes: Depending on responses (to a, b, or c above) –ask questions related with intervention components that were accessed:

What did you think of a? b? c? Probes: Were these **easy to access**? Were there certain things that made it easy to access the program? Were there things that made it harder to access the program? (Probe: If participants or not discuss-'impact of time' to engage with components of program). (If not (easy to access)- why not, what made it difficult to access? please explain)

- Were there things that you particularly liked, or thought were useful? (if needed probe: Why do you say that)
- What did you like least about these components? (probes: number / frequency of messages- would have preferred more or less of ?
- What did you find less useful? (if needed probe: Why do you say that)
- Do you have any suggestions on how we could improve the design of these components?
- Do you have any suggestions on how we could improve the delivery of these components?
- How would you like to access this type of training in the future? (Probe: e.g., by what medium- note preferences for in-person / digital?
- Where would you like to see this sort of information delivered so that members in your community can access it easily? (prompt on social media? FB?)
- What is your perception of attitudes and beliefs towards responding to cardiac arrest emergencies in your community?
- Do you believe the FirstCPR program has been effective in encouraging community members to seeking training in CPR? (if needed probe: Why do you say that)
- Do you believe the FirstCPR program has been effective in encouraging community members to seeking training in AED use? (if needed probe: Why do you say that)
- Do you believe the FirstCPR program has been effective in changing community members' attitudes about responding to a cardiac arrest emergency? (if needed probe: Why do you say that)
- What do you think is the best method to spread FirstCPR's messages among members of your community? (if needed probe: Why do you say that)
- What do you think is the best method to encourage community members to learn about responding to cardiac arrest emergencies? (if needed probe: Why do you say that)

- What do you think is the best method to encourage community members to be prepared to respond to cardiac arrest emergencies?
- Or What do you think are good approaches to conduct similar health education programs in your community?

CLOSE: Are there any other comments you would like to make before we end?

3. Nominative labelling to summarise intervention implementation

Table S1: Nominal categories 0 to 4 summarised intervention delivery to organisation and member engagement

Category	Nominative label summarising intervention delivery and member engagement	Allocation criteria
**4**	Fully delivered, some engagement	All four intervention components were delivered at the organisation / to members & >20% or > n=30 members engaged (with one or more components)
**3**	Fully delivered, low engagement	All four intervention components were delivered & ≤20% or ≤ n=30 members engaged
**2**	Partially delivered, some engagement	Some of the intervention components were delivered & >20% or > n=30 members engaged
**1**	Partially delivered, low engagement	Some of the intervention components were delivered & ≤20% or ≤ n=30 members engaged
**0**	Not delivered or Withdrew	None of the intervention components delivered OR Withdrawal / discontinued participation

4. Organisation recruitment

Figure S2: Screening and recruitment by organisation location (Urban / Regional area)

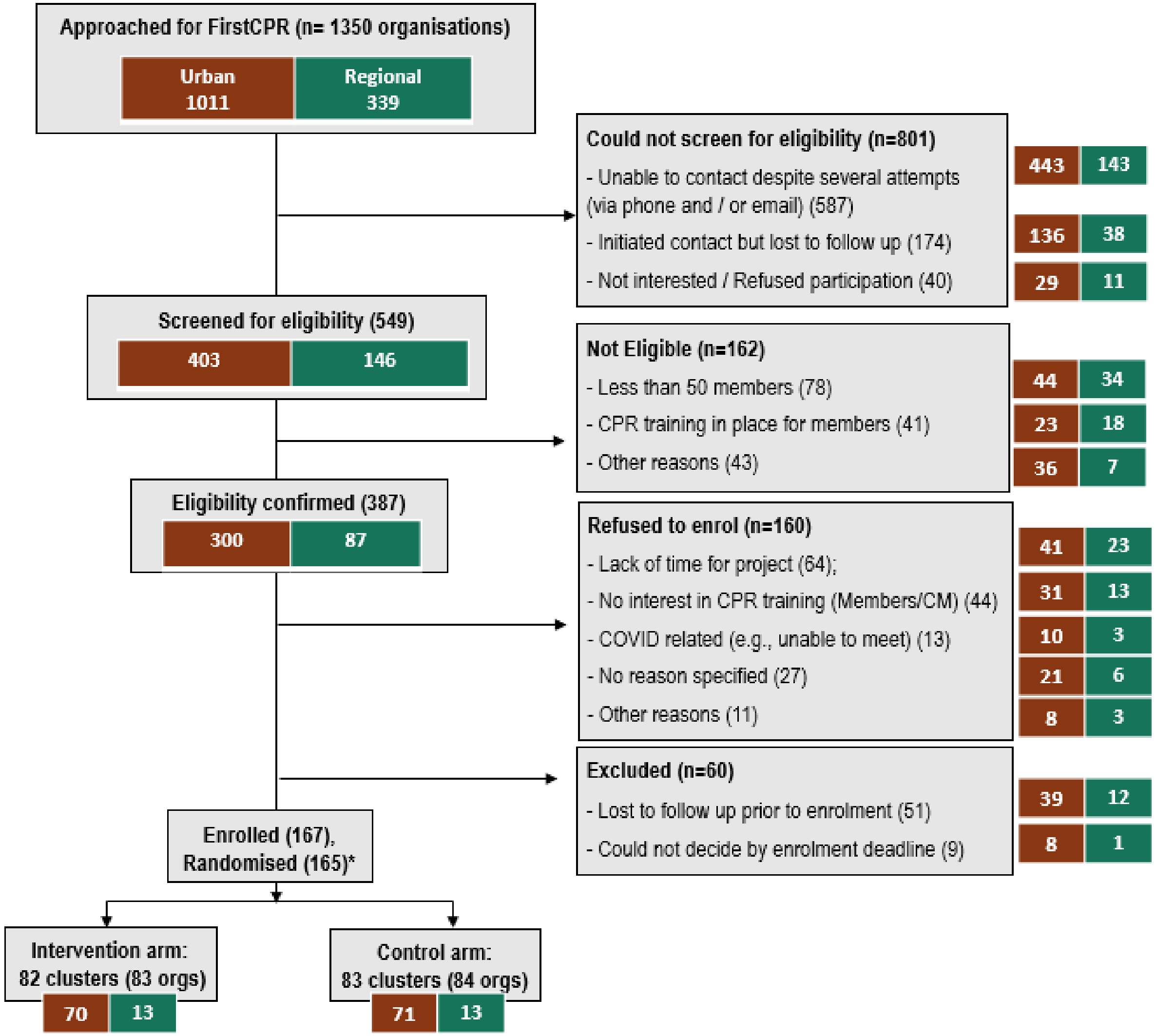

Table S2: Reasons for organisation ineligibility

<table>
<tr><th>Main reasons* N=162
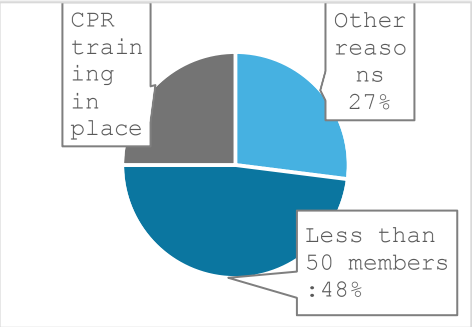
</th><th>Examples of reasons/quotes provided by organisation liaison (via phone/email)[‡].</th></tr>
<tr><td>1. Less than 50 members (n=78)</td><td>"We would be interested but unfortunately don't have 50 members". Church; Regional location
"The club has low membership numbers not since COVID restrictions eased and can no longer reach 50 members" – Netball club, Urban location</td></tr>
<tr><td>2. CPR training already in place (n=41)</td><td>"We would have been interested but has CPR program in place already as all coaches and staff have to do, and promoted to members also" – Tennis centre, Urban location
"Received CPR training for members 2 months ago through [accredited training organisation] and participation of members was quite high, and they will be receiving ongoing training"- Rotary club, Regional location</td></tr>
<tr><td colspan="2">3. OTHER reasons (n=43) listed below</td></tr>
<tr><td>Club dissolved, mostly COVID-related (n=14)</td><td>"Club closed down due to COVID otherwise would have opted in" – Sports org (District netball club); Urban location</td></tr>
<tr><td>Unable to support all steps (e.g., difficult to communicate with members digitally) (n=7)</td><td>"We mainly have seniors here and means potential difficulties in digital communication"- Church group, Regional location</td></tr>
<tr><td>Study cannot support language needs (n=2)
i.e., Language not one of the four languages offered by the study.</td><td>"We would need information in Dari- our members can barely understand where to go for training and it's just not the time to reach out to our members for something like this which is so complicated. They are in distress at the moment - with all that's going on in Afghanistan and the lockdown etc- it's a crisis situation (note: He noted the timing of such research may also be inappropriate due to other contextual factors such as Afghanistan civil crisis with US military leaving and Taliban takeover plus COVID lockdown"- Soccer club, Urban location</td></tr>
</table>

**No base of operations (n=4)**	*"Do not have a venue or location where people gather"*- Social online multicultural community, Urban location
**No physical address in study area (n=4)**	*"The business has moved and is no longer located within study area"* – local business, Regional location
**Significant cross-membership and unable to merge (n=2)**	*"Most of the senior members and parents of junior members are already a part of a leagues club nearby that is already enrolled"*- Cricket club, Urban location
**Mostly a service-based organisation without stable membership (n=5)**	*"We are a small community care mostly*- for service and we offer a cup of tea /food and for them to have a sit down - *not a conventional church ; just give out food to people - like a tiny shop…We are based in a location where people are not tech savvy, language problems, non-English speaking and mostly seniors"*- Church group, Urban location
**Difficulty in delivering intervention due to renovations (n=1)**	*"…cannot be part of the program due to renovations at the church"* – Church group, Urban location
**Peak body or umbrella organisation for other clubs (n=4)**	*"…just an administration office and so we would need to contact the churches individually"*- Church group head office, Urban location

**Codes elucidated from phone log/email communication with organisation liaison person/s ‡Quotes are mostly verbatim (and sometimes summary information) of conversations recorded by members of the study team based on phone conversations and sometimes email communication is copied over.*

Table S3: Reasons for organisation refusals

Main reasons: N=160	Examples of reasons/quotes provided by organisation liaison
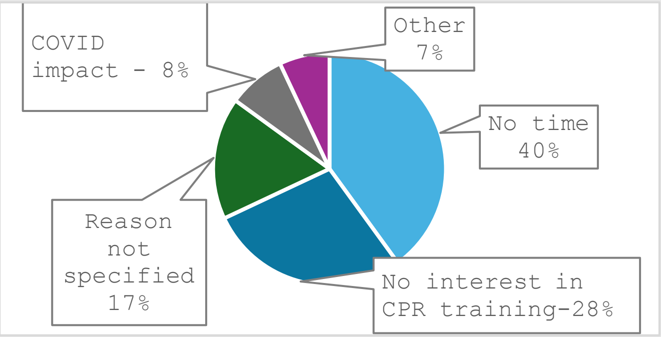	
**1. No time/capacity at organisation to facilitate this program (n=64)**	*"It's a great initiative, but timing is not great for them so will not opt in"* - Golf club, Regional location.<br><br>*"Not interested in participating anymore as we are in the off season"* - Football cub, Urban location.
**2. Committee members and/or organisation members not interested in CPR training (n=44)**	*"Committee member raised the program at virtual meeting of the congregation, and they debated for some time, one person had seen a heart attack, but the rest of the congregation said others tend to be trained in CPR so overall no interest with vast majority of members"* - Church group, Urban location.<br><br>*"Members are only interested in full first aid course not CPR only"*- Church group, Regional location.
**3. COVID-related uncertainties and impact (e.g., unable to meet due to restrictions or lockdown (n=13)**	*"Due to Covid restrictions last 4-5months - things have been a bit disruptive and not meeting members more than once every 4-5months. Doubt they would be interested in anything like this at the moment even if the reach is digital but try us again sometime end May and we will see what we can do"*- Community services group, Urban location<br><br>*"Admin staff who indicated that the priest will not be interested in anything like that especially given the lockdown - try next year when things have opened up"*- Church group, Urban location.

**4. Reason not specified (n=28)**	*"Church just not interested, no reason given"* – Church group, Urban location.<br><br>*"After discussion with Management, we have decided not to proceed with the project"* – Social RSL/Leagues type social club, Urban location.
4. OTHER reasons (n=11) listed below	
**Already have enough CPR training in place (n=7)**	*"Not interested as we have our own CPR training program at site - where they come in and offer it to all members"* – Community care services, Urban location.<br><br>*"Small group and most people have already done training and know CPR so not interested"*- Church group, Regional location.
**Other (multiple reasons cited) (n= 4)**	*"Won't be participating, don't have our own place (meet in a school hall), would be difficult to get everyone together in, they don't feel like they have a lot of members. A lot of their members get first-aid training in their workplaces"*- Football club, Urban location.<br><br>*"[will not be part of the study for] "being in a middle of some personal issues"*- Netball club, Urban location.

**Codes elucidated from phone log/email communication with organisation liaison person/s; ‡Quotes are mostly verbatim (and sometimes summary information) of conversations recorded by members of the study team based on phone conversations and sometimes email communication is copied over.*

5. Intervention implementation at intervention clusters

Figure S3: Summarising intervention delivery and uptake by organisation type using the scoring system

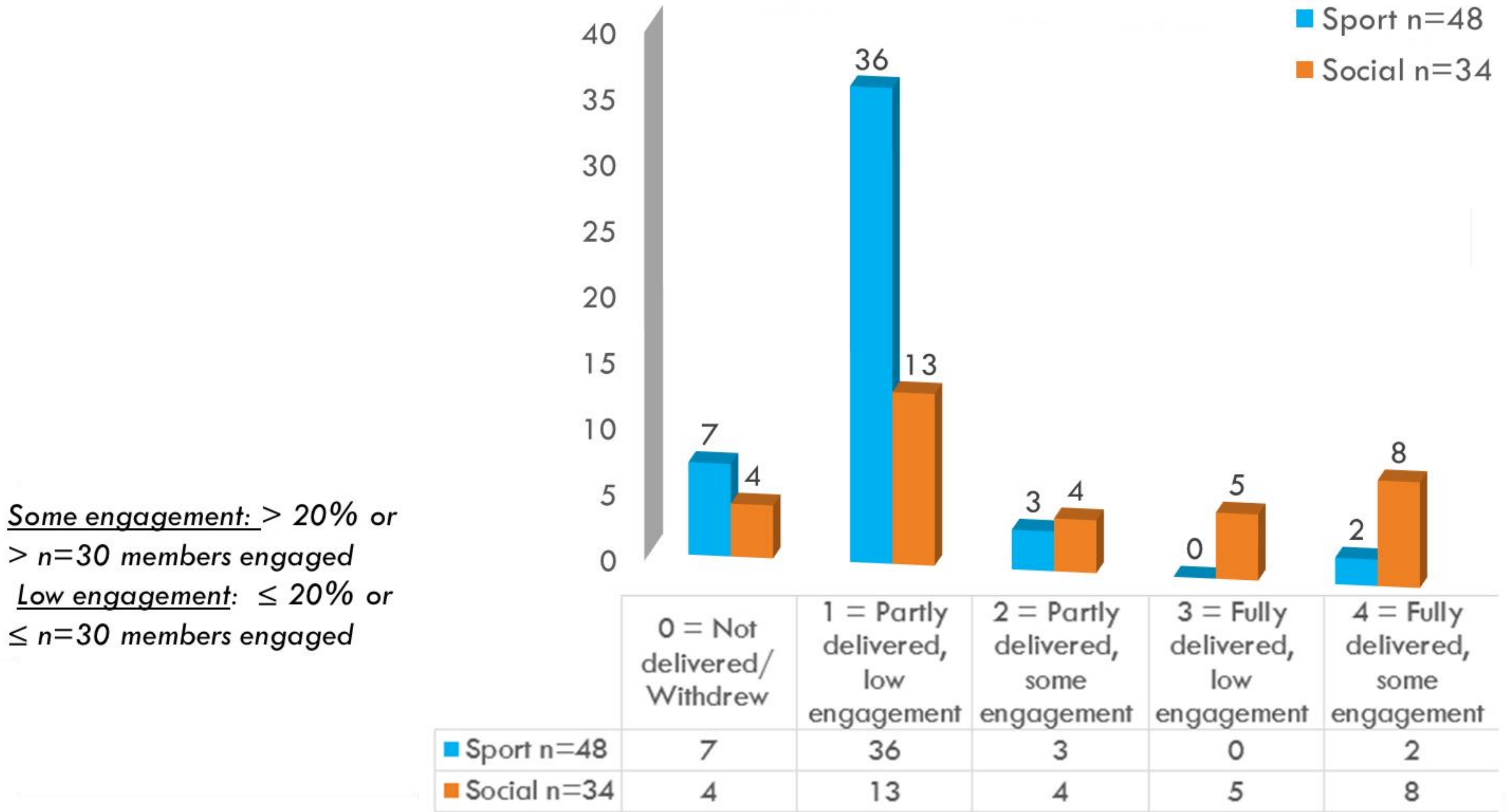

	0 = Not delivered/ Withdrew	1 = Partly delivered, low engagement	2 = Partly delivered, some engagement	3 = Fully delivered, low engagement	4 = Fully delivered, some engagement
Sport n=48	7	36	3	0	2
Social n=34	4	13	4	5	8

Our scoring system categorised organisations with scores of 4 or 3 as those that received all components of the intervention and scored highest on delivery + engagement. There was, however, a significant variability across similar sports or social groups as depicted in Figure 3, and a SANKEY diagram in the Supplementary section of the FirstCPR manuscript [11]. This Figure previously appeared in Munot et al, 2025. FirstCPR: A pragmatic community organisation-based cluster randomised trial to increase community training and preparedness to respond to out-of-hospital cardiac arrest. Resus plus 2025 Vol. 23 Pages 100949. *Reproduced here under the terms of the Creative Commons Attribution License (CC BY).*

Table S4: Features of committee liaisons (interviewees) and focus group discussion participants

ID	Data collection	Age group	Gender	Organisation type	Organisation Size (~number of members)	Organisation location
P1	Interview	70-75	Male	Social: Multicultural	Moderate (n~800)	Urban
P2	Interview	n/a	Female	Leagues club	Large (n~3000)	Urban
P3	Interview	65-70	Male	Faith-based organisation	Small (n~50)	Urban
P4	Interview	65-70	Female	Aquatic centre	Moderate (n~500)	Regional
P5	Interview	70-74	Female	Faith-based organisation	Small (n~123)	Regional
P6	Interview	55-60	Male	Social: Multicultural	Large (n~1050)	Urban
P7	Interview	40-45	Male	Faith-based organisation	Small (n~57)	Regional
P8	Interview	n/a	Male	Sport	Small (n~100)	Urban
P9	Interview	60-65	Male	Faith-based, Multicultural	Large (n~630)	Urban
P10	Interview	50-55	Male	Faith-based, Multicultural	Large (n~7019)	Urban
P11	FGD	45-50	Male	Sport	Large (n~800)	Urban
P12	FGD	65-70	Female	Social: Multicultural	Small (n~70)	Urban
P13	FGD	75-80	Male	Social: Multicultural	Large (n~300)	Urban
P14	FGD	40-45	Female	Sport	Large (n~800)	Urban
P15	FGD	40-45	Female	Social	Large (n~3500)	Urban
P16	FGD	45-50	Male	Faith	Small (n~100)	Urban
P17	FGD	45-50	Female	Sport	Large (n~1200)	Urban
P18	FGD	50-55	Male	Sport	Large(n~250)	Urban
P19	FGD*	55-60	Female	Social: Multicultural	Large (n~350)	Urban
P20	FGD*	65-70	Female	Social: Multicultural	Large (n~350)	Urban
P21	FGD*	65-70	Female	Social: Multicultural	Large (n~350)	Urban
P22	FGD*	60-65	Female	Social: Multicultural	Large (n~350)	Urban

P23	FGD*	60-65	Female	Social: Multicultural	Large (n~350)	Urban
P24	FGD*	55-60	Female	Social: Multicultural	Large (n~350)	Urban

*Note: Interview participants represented a spectrum of organisational experiences, ranging from full delivery of intervention components with varying levels of member engagement to no intervention delivery or engagement. *Vietnamese facilitator.*

Table S5: Features of intervention organisations that withdrew from FirstCPR

**Org Type**	**Location**	**Size: Number of members**	**Withdrawal timepoint post-enrolment**	**Intervention delivery to members prior to withdrawal**	**Reason/s**
Faith –Church (Multicultural)	Urban	210	5 months	*12 members consented to digital messages	Committee members do not have time
Sport – Gym	Urban	415	5 months	*2 members consented to digital messages	Believe program may not bring benefit; Promoting a survey does not align with their brand standards.
Sport – Soccer	Urban	900	6 months	*2 members consented to digital messages	Committee members do not have time.
Sport – Soccer	Urban	900	11 months	None	Limited time of committee members; Committee has not filled all positions (therefore significant additional pressures for those on the committee)

Org Type	Location	Size: Number of members	Withdrawal timepoint post-enrolment	Intervention delivery to members prior to withdrawal	Reason/s
Sport – Rugby	Urban	120	10 Months	None	Time constraints; other higher priorities, flood damage to grounds, COVID forcing members into isolation; members taking the opportunity to be able to travel interstate / internationally
Faith – Church	Regional	50	<1 month	None	Not able to fulfil the requirements of the study (minimum members contactable) and so chose to withdraw.
Sports-Other	Regional	100	11 months	None	Personal health issues restricted committee liaison to facilitate study activities, club restructure post-COVID and uncertainty with operations
Sports-Tennis	Urban	300	11 months	*7 members consented to digital messages	Committee liaison indicated difficulty to drum up interest participation. Most activities are after work hours, held outdoors and members just want to come and play tennis. However, FirstCPR did initiate the organisation to purchase an AED for the club and organisation committee members have completed First aid/CPR course.

**Study link was circulated to members*

Table S6: Participant recommendations and suggestions for future community-based training

Suggestion codes	Representative quotes
Central hub of information	"… I'd love a cheat sheet, along that same sort of theme. I'm sure there's a double-sided sheet that could…could then summarise all the key points to just have…tucked away somewhere that's…that's readily accessible." - P18<br><br>"Is it available on a website as a-…fact 1,2,3,4, like, so, if there were...like 15 or 20 of them, videos, messages, whatever. Are they all in a concise, you know, website page that people can go back and click on them" - P17
Find a captive audience	"you could even do a marketing thing with a QR code, you know, a…a simple size QR code and…and stick them on the insides of trains and buses, it says 'got 10 minutes? Learn CPR now" P17<br><br>" …how about taking it up a level to, you know, the rugby league teams and the AFL teams whose club memberships are by the thousands…. to go along to [club central/headquarters] … [celebrity sports person] may come here as well … I think it might attract a few more people as well because it's coming through their select club membership, get them an opportunity to go to their local club that they might not have been to their HQ before…" - P17<br><br>"… soccer stars coming to the soccer club, to get more people there and…do the thing there. I mean that would be the dream, wouldn't it, to get everyone on board, that way-…they're going there for another purpose but while they're there…captive audience." - P17
More AED content	"I don't know whether that's part of your research, best practice AED positioning" - P18<br><br>"I see AED's everywhere now, and…and I-…yeah, whenever I'm wandering in and out of places ...I notice them, I know what they are. Probably the one thing that could be added as part of the…of the training, is just some guidance in terms of accessing them because they're obviously not readily open" -P18
Boost participation by raising awareness of the importance of learning / refreshing skills in advance of organising the event	"I think I put it on social media at one point as well. Yeah, and social media probably is pretty good. But at the same time, unless it's a paid post, it doesn't…. A lot of people may not see it" – P2<br><br>"also spreading awareness to others because this is also a cascading effect. Because the husband is coming, we want his wife to come, you know, we want other people to come. So, it's that they really motivate, they've actually will bring other, you know, members who may not actually be not necessarily be as engaging." -P9
Tailoring messaging to the target audience	"(In our culture) saving somebody's life is considered...as if you've saved humanity.... whoever saves one life, as if he saved mankind or humanity. ....if you are in a position to save someone's life, then you should. … those bits can be helpful sometimes, having that culturally appropriate messaging....well, this is what your scriptures are saying, I think (can be) engaging more then what we are doing is (as it) actually identifies with your values …so people might actually become more committed to do it and not just about attending the program, but also spreading awareness to others because this is also a cascading effect (in the community)" – P9.

Box 1. Questions and comments by members in the audience at the interactive information sessions

Questions/comments related to arrest recognition

- How do you make sure they are in cardiac arrest? How can you be sure they haven't just fainted?
- How can you the difference when someone has fainted but is not in cardiac arrest?
- What if you do CPR on someone who doesn’t need it?
- Difference between heart attack and cardiac arrest?
- So, what is a heart attack?
- Are there any devices you can keep at home to help address a cardiac arrest?

Questions/comments related to CPR

- Is it worth performing CPR if they've been unconscious for 5 minutes? (concern about the impacts of permanent brain damage)
- Fear of breaking ribs due to compression
- How to identify where to place hands on the chest for compressions
- Is a child allowed to perform CPR?
- When you give someone mouth-to-mouth CPR, what does that do to them?
- Can you predict or have a test to confirm if a cardiac arrest will happen?
- What does CPR do?
- An 11-year-old asked if they could do CPR, given their age?"
- How long should a bystander perform CPR for?
- When should you give breaths - does it provide more benefit?
- What would be the difference if you don't do breaths, would be the same as hands only?
- Would it cause harm if you start CPR in someone who is not in CA?
- What happens if we push too deep?
- How long I need to keep doing CPR?
- What to do if the person becomes conscious while doing CPR?

Questions/comments related to AEDs

- AED Pad placement - Adults, children
- Locating AEDs in an emergency
- Why can't we keep shocking with AED: why AED + 2min CPR- then AED again cycle

- What is a defibrillator?
- Can you use the AED for a child?
- Can you explain pad placement for a child?
- Application of an AED/pads in someone with a pacemaker?
- Is there any risk for the person who is applying the shock using the AED?
- What does the shock do?
- How much does an AED cost, and what about maintenance/battery?
- Why are the pads positioned this way? (Not directly on the heart area)
- How long do you wait between starting cpr and putting the defib on?
- Why can’t you use the AED when a person is in flatline? I.e. why don’t AEDs shock a person when they’re in flatline?

Miscellaneous questions/comments

- Can a healthy person have a heart attack?
- Do we have to remove clothing from a woman if they have a heart attack?
- Can you give CPR to someone who has an implantable device
- Does doing CPR on a real person feel the same as it feels when compressing a mannikin?
- What about Consent?
- Can cardiac arrest be detected with a smart watch/Fitbit
- How long does it usually take for the victim to 'come back'?
- What happens if they start vomiting or putting blood in their mouth? Should I wash it?
- Why do 80% of cardiac arrests occur in the home? Why in that location?
- Can you not just take the person to the hospital?
