# Supplementary material for: Process evaluation of a basic life support educational intervention (FirstCPR cluster randomised study) delivered at community organisations in New South Wales, Australia
Source: BMJ Open. 2026 Apr 7;16(4):e113343. doi: 10.1136/bmjopen-2025-113343 (PMC13064210; doi:10.1136/bmjopen-2025-113343)
Supplement: Supplementary file 2 [file bmjopen-2025-113343.reviewer_comments.pdf]

PEER REVIEW HISTORY

BMJ Open publishes all reviews undertaken for accepted manuscripts. Reviewers are asked to complete a checklist review form and are provided with free text boxes to elaborate on their assessment. These free text comments are reproduced below.

ARTICLE DETAILS

Title (Provisional)

Process evaluation of a basic life support educational intervention (FirstCPR cluster randomised study) delivered at community organisations in New South Wales, Australia

Authors

Munot, Sonali; Bauman, Adrian; Bray, Janet; Redfern, Julie; Angell, Blake; Kancijanic, Diane; Rock , Zoe; Semsarian , Christopher; Jennings , Garry; Coggins , Andrew; Denniss, Alan Robert; Chow, Clara

---

VERSION 1 - REVIEW

**Reviewer**	**1**
**Name**	**Myklebust, Helge**
**Affiliation**	**Laerdal Medical Cooperation, Strategic Research**
**Date**	**19-Dec-2025**
**COI**	**I work as Director of Research in Laerdal Medical, a company who help partners design and deliver CPR training solutions. However, Laerdal Medical's work needs to be informed by research in order to be effective.**

This is a comprehensive study that adds new insight about community CPR training from participating organisations perspective and from individual learners perspectives. The Aim of the study was to evaluate a) Enrollment and study participation and b) implementation including reach, uptake and engagement. (Pg 4, lines 21-23). The result section should be organized in the same manner, using the same terminology. Now it is difficult to see is/how the study design has evaluated the study aims. Triangulation is mentioned as a method for analysis, but not described in such a way that it can be replicated. The use of purposeful, maximum variation sampling strategy is mentioned but without sufficient detail to understand how it was done. Pg 9, Line 7. Title says Patient and should be deleted. Pg 19 Line 7. Title says ..through work - but the following text does not describe work. Suggest to delete "through work". The Conclusion sections should be shortened and structured according to the study aims. Limitations must include the risk of selection bias as only 160 of 387 eligible organizations participated.

Reviewer 2

Name Wittig, Johannes

Affiliation Aarhus Universitet, Department of Clinical Medicine

Date 27-Jan-2026

COI Volunteer activities:
- European Resuscitation Council (ERC) Science and Education Committee Advanced Life Support (ALS)
- Young ERC Committee
- International Liaison Committee on Resuscitation ALS Task Force member

Research funding:
- Laerdal Foundation

Thank you for the opportunity to review this mixed-methods study which aimed to provide a process evaluation for a basic life support (BLS) educational intervention delivered to community organizations within a cluster randomized trial in Australia.

Effective educational interventions to improve BLS competencies and preparedness to respond to a cardiac arrest among the general population are crucial to improve outcomes after cardiac arrest. Detailed investigation, exploration and understanding how these interventions were implemented and engaged with by trial participants allows for identification of barriers and facilitators which may inform future trials and contextualize the results of the FirstCPR trial. This mixed-methods study provides valuable insights that may advance implementation, refinement and investigation of similar BLS interventions on a community level. The paper is overall well written and is likely of value to the scientific community engaged in public health and response to out-of-hospital cardiac arrest.

However, I have several suggestions that I would kindly ask the authors to consider.

References to specific sections in the text are written as P(age)XL(ine)Y.

Abstract

P2L9: It may not be clear to all readers what is meant by an “uptake metric”. Consider if alternative wording or brief elaboration would be helpful.

P2L11-12: Please elaborate on what is meant with “data triangulation”. I assume this references multiple data sources to explore/confirm themes.

Please report categorical variables as counts and proportions n(%).

Strengths and Limitations

P3L20-21: Consider if this is truly a strength or limitation of this study or a unique challenge that emerged from the trial context.

Introduction

No comments.

Methods

P5L6: Please provide a reference to the FirstCPR trial indicating where it is described.

Please report categorical variables as counts and proportions n(%).

Please provide measurements of variability for mean (standard deviation) and median (first quartile; third quartile).

Please note that the interquartile range is technically a single value and is commonly confused with the first quartile and third quartile (Q1; Q3).

P7L5-11: I assume this is not a validated system. If this is correct, please consider discussing this limitation briefly in the Limitations section.

Results

The results section is lengthy which is an indication of the richness of the data that was collected. However, in its current state it is difficult to follow and at times the overall structure is confusing which risks that key findings are overlooked. Please consider whether the number and levels of subheadings can be reduced, whether all findings need to be discussed in this detail and please consider what the key findings are that need to be conveyed here and what information could also be made available in a supplementary results section.

Additionally, it is currently difficult to distinguish between the various sources of information/data which makes interpretation challenging. I would suggest uniform wording to introduce findings from another source and/or separation within a paragraph.

Please ensure that reporting of results is kept separate from discussion of results.

P9L2-5: Please consider discussing this in terms of limited generalizability within the limitations section of the manuscript.

P11L10: Please remain specific when describing reasons for withdrawal or loss to follow-up.

P12L22-24: Please provide information on the population that responded to the survey and whether there is information on survey recall available. Please consider discussing the limitations and potential bias arising from voluntary surveys and potentially limited recall in the limitations section.

P14L5: Please avoid the numbering of subheadings as this may lead to confusion with the two overall result sections.

P14L18-19: I am unsure if this is speculation or results being reported. Please clarify this sentence and if needed move it to discussion sections.

P14L21-22 and P15L1-2: This seems to be interpretation or speculation. In any case, this would require data that can show this effect on participation levels. The current statement can be used as a speculation in the discussion but should still be supported by data.

P15L6: The sentence seems to be more appropriate for a discussion section.

Discussion

P25L17-18: While it is fine to contextualize limitations, please avoid mentioning strengths in the limitations section.

Conclusions

The conclusions section is substantially too long, and I would suggest to the authors to focus on key aspects they wish to convey that are supported by the results of this study. Any other parts can be integrated into the discussion.

I hope my comments help to further improve the authors' valuable work.

VERSION 1 - AUTHOR RESPONSE

Reviewer: 1
Mr. Helge Myklebust, Laerdal Medical Cooperation
Comments to the Author:
This is a comprehensive study that adds new insight about community CPR training from participating organisations perspective and from individual learners perspectives.

Many thanks for your thoughtful and very constructive comments which have helped strengthen the manuscript. We have addressed each point carefully and revised the manuscript accordingly. Detailed, point-by-point responses are provided below, with changes indicated in the revised manuscript with page and line numbers (**pg and line numbers correspond to the *tracked version;*** *a clean version* is also attached).

The Aim of the study was to evaluate a) Enrolment and study participation and b) implementation including reach, uptake and engagement. (Pg 4, lines 21-23). The result section should be organized in the same manner, using the same terminology. Now it is difficult to see is/how the study design has evaluated the study aims.

The Results section has now been considerably restructured to read more simply and better align with the Aims stated in the Introduction. Headings and subheadings have been reduced and revised, and numbering simplified throughout for better clarity and for easier reading flow. They link better with prespecified study aims evaluating *organisation enrolment and factors influencing study participation (Aim a) and Intervention implementation presented under the domains of reach, uptake, and engagement (Aim b).*

Please see the ***Results section- starting on Page 10, Line 19*** - reorganised now for better flow and readability and link between aims, data sources and related findings as noted below:

Aligned with Aim 1 we begin by presenting organisational enrolment and determinants of study participation. We then address Aim 2 by reporting intervention implementation across intervention clusters, organised by reach, uptake, and engagement. Quantitative data are presented alongside qualitative findings from interviews, focus group discussions, researcher observations and field notes, and liaison communications. See Tables 1-2 and Supplementary tables for illustrative quotes.

ORGANISATION ENROLMENT AND FACTORS INFLUENCING STUDY PARTICIPATION

Organisation screening, eligibility, and enrolment (quantitative, supported by Figure 2)

Barriers to organisation enrolment/participation

Enablers to organisation enrolment/participation

INTERVENTION IMPLEMENTATION (REACH, UPTAKE AND ENGAGEMENT)

Reach (distribution/exposure to intervention components)

Uptake (opt-in and in-person attendance)

Engagement (interaction with components and cluster-level variability

Participant feedback on intervention (acceptability and suggestions)

Factors influencing intervention implementation and member engagement (Table 2)

-Contextual /extraneous factors

Subthemes listed (bold and italic, no numbering)

-Intervention design and delivery-related factors

Subthemes listed (bold and italic, no numbering)

Triangulation is mentioned as a method for analysis, but not described in such a way that it can be replicated.

We acknowledge that the use of the term ‘triangulation’ may be somewhat misleading, as no formal triangulation protocol was employed. Instead, multiple data sources were used to explore and confirm themes. Quantitative and qualitative datasets were analysed separately, and findings were subsequently considered together during interpretation. This approach aligns more closely with a multi-method design. We have revised the manuscript to describe the study as a ‘multi-method’ process evaluation and to clarify that the findings are presented in accordance with the UK Medical Research Council (MRC) process evaluation framework. Terminology in the Abstract and Methods sections has been updated for consistency.

Pg 2 Lines 6 – 7; Abstract/Methods*: The study used a multi-method process evaluation. Data were collected … and* ***lines 14 – 17.*** *Qualitative and quantitative data were analysed separately and subsequently interpreted collectively to contextualise implementation patterns and identify barriers and enablers that influenced trial successes and failures.*

Pg 3 Lines 23-24l Pg 4 lines 1-2 Strengths and Limitations section *removed the word 'triangulation':*

- A multi-methods process evaluation enabled a nuanced understanding of intervention implementation across diverse community contexts
- The use of multiple data sources enabled the capture of diverse perspectives from participants and facilitators

Pg6 Lines 22 – 25 and Pg 7 Lines 1-7: Methods/Data sources:
Process evaluation measures were planned a priori to explore the intervention strategy, design and delivery, potential mechanisms of action, and contextual factors influencing participation and uptake [16]. A multi-method approach was used, with data collected via study team observations and field notes, web analytics, surveys, semi-structured interviews, and focus group discussions. Quantitative and qualitative datasets were analysed separately. Findings from the various data sources were subsequently considered together during interpretation to provide a comprehensive understanding of intervention delivery, program mechanisms, member reach and engagement, and barriers and enablers to participation [18,19]. Quantitative process indicators and qualitative themes are presented narratively, with qualitative findings used to contextualise observed quantitative patterns.

Pg 9 Lines 22 – 25; Pg 10 lines 1 - 5 -: Methods/Data analysis:
Findings and patterns emerging from the analysis of data from multiple sources were considered together during interpretation and are presented in alignment with the UK Medical Research Council (MRC) process evaluation framework [15,16]. Interpretation was guided by the pre-specified aims and MRC framework domains. The framework supports process evaluation across three areas: examining implementation (including delivery, structure, fidelity, and reach), exploring mechanisms of impact (how the intervention works), and considering contextual factors influencing implementation

The use of purposeful, maximum variation sampling strategy is mentioned but without sufficient detail to understand how it was done. Pg 9, Line 7.

We have now revised the Methods to provide greater clarity and more detail on the sampling frame and the variation variables used to guide selection (e.g., organisation type, metro/regional location, and implementation/engagement level). We also clarify the stopping criterion (sampling continued until thematic sufficiency/saturation was reached and within the constraints of recruitment).

See Pg 7 Lines 24 -25; Page 8 Lines 1 – 8: Methods/Data Sources: *A purposeful, maximum variation sampling strategy was used for liaison interviews to capture a range of perspectives across key characteristics. Liaisons were approached to maximise variation by choosing diverse organisation types (e.g., social, multicultural, faith-based groups, sports) and locations (metro and regional) and where feasible, by key demographic characteristics (gender, age group). Liaisons' recruitment was iterative with invitations directed towards maximising under-represented organisation types, locations, and participation levels*

(including minimal or no member engagement) as feasible. The final mix of participants is summarised in Suppl Table S4.

Title says Patient and should be deleted. Pg 19 Line 7.

As per your suggestions we had deleted the word ‘Patient’ but we were asked to reinsert this by the Editor as this a standard subheading for BMJ.

Title says ..through work - but the following text does not describe work. Suggest to delete "through work".

We have deleted the phrase “through work” ***(Pg 24 line 2)***

The Conclusion sections should be shortened and structured according to the study aims.

We have substantially shortened the Conclusions section (*now 147 words*) to focus only on the key take-home messages directly supported by the results of this process evaluation. The revised Conclusions are explicitly structured to reflect the study aims, and broader implications beyond the primary findings have been consolidated within the Discussion to improve clarity and maintain appropriate separation between interpretation and concluding statements. **Please see the updated *Conclusion section on Page 32***

Limitations must include the risk of selection bias as only 160 of 387 eligible organizations participated.

We have revised the Limitations section to explicitly acknowledge potential selection bias arising from the proportion of eligible organisations that participated and to note implications for transferability, particularly for time-poor organisations or those without an active liaison/champion. We also note attrition over follow-up and its potential to influence observed implementation patterns.

Pg 31 lines 19 - 24: Limitations section: *There is also a risk of selection bias and limited generalisability as only 167 of 387 eligible organisations participated. Those who took part may have had greater capacity, motivation or leadership support for BLS training than those who declined or were not retained, limiting transferability of findings to time-poor organisations or those with limited facilitation capacity. Attrition over follow-up, with additional dropouts before the 12-month assessment, may also have influenced observed implementation patterns.*

Reviewer: 2

Dr. Johannes Wittig, Aarhus Universitet
Comments to the Author:
Thank you for the opportunity to review this mixed-methods study which aimed to provide a process evaluation for a basic life support (BLS) educational intervention delivered to community organizations within a cluster randomized trial in Australia.

Effective educational interventions to improve BLS competencies and preparedness to respond to a cardiac arrest among the general population are crucial to improve outcomes after cardiac arrest. Detailed investigation, exploration and understanding how these interventions were implemented and engaged with by trial participants allows for identification of barriers and facilitators which may inform future trials and contextualize the results of the FirstCPR trial. This mixed-methods study provides valuable insights that may advance implementation, refinement and investigation of similar BLS interventions on a community level. The paper is overall well written and is likely of value to the scientific community engaged in public health and response to out-of-hospital cardiac arrest.

However, I have several suggestions that I would kindly ask the authors to consider. References to specific sections in the text are written as P(age)XL(ine)Y.

We are grateful to the reviewer for their review and very constructive feedback. These comments have been valuable in strengthening the manuscript. Each point has been considered in full and addressed below with corresponding revisions in the manuscript (Page and line numbers correspond to the *tracked version,* and a *clean version* is also attached).

Abstract
P2L9: It may not be clear to all readers what is meant by an "uptake metric". Consider if alternative wording or brief elaboration would be helpful.
We agree that 'uptake metrics' may be unclear as stated in the abstract. This has been revised to 'implementation measures', and we have added a brief clarification of what uptake refers to (e.g., opt-in to digital messages and attendance at training sessions).

Pg 2 Lines 8 -11 Abstract: *These sources captured participation patterns and implementation measures (delivery, reach, uptake and engagement - opt-in to digital messages, attendance at training sessions), as well as reasons for refusals and withdrawals.*

P2L11-12: Please elaborate on what is meant with "data triangulation". I assume this references multiple data sources to explore/confirm themes.
Multiple data sources were used to explore and confirm themes. However, the term 'data triangulation' may be somewhat misleading, as no formal triangulation protocol was used. Instead, quantitative and qualitative datasets were analysed separately, and findings were subsequently considered together during interpretation. This approach aligns more closely with a multi-method design. We have revised the manuscript to describe the study as a 'multi-

methods' process evaluation and clarified that findings are presented in accordance with the UK Medical Research Council (MRC) process evaluation framework. Terminology in the Abstract and Methods sections has been updated for consistency.

***Pg 2 Lines 6 – 7; Abstract/Methods**: The study used a multi-method process evaluation. Data were collected … and **lines 14 – 17.** Qualitative and quantitative data were analysed separately and subsequently interpreted collectively to contextualise implementation patterns and identify barriers and enablers that influenced trial successes and failures.*

***Pg 3 Lines 23-24l Pg 4 lines 1-2 Strengths and Limitations section** removed the word 'triangulation':*

- A multi-method process evaluation enabled a nuanced understanding of intervention implementation across diverse community contexts
- The use of multiple data sources enabled the capture of diverse perspectives from participants and facilitators

Pg6 Lines 22 – 25 and Pg 7 Lines 1-7: Methods/Data sources:
Process evaluation measures were planned a priori to explore the intervention strategy, design and delivery, potential mechanisms of action, and contextual factors influencing participation and uptake [16]. A multi-method approach was used, with data collected via study team observations and field notes, web analytics, surveys, semi-structured interviews, and focus group discussions. Quantitative and qualitative datasets were analysed separately. Findings from the various data sources were subsequently considered together during interpretation to provide a comprehensive understanding of intervention delivery, program mechanisms, member reach and engagement, and barriers and enablers to participation [18,19]. Quantitative process indicators and qualitative themes are presented narratively, with qualitative findings used to contextualise observed quantitative patterns.

Pg 9 Lines 22 – 25; Pg 10 lines 1 - 5 -: Methods/Data analysis:
Findings and patterns emerging from the analysis of data from multiple sources were considered together during interpretation and are presented in alignment with the UK Medical Research Council (MRC) process evaluation framework [15,16]. Interpretation was guided by the pre-specified aims and MRC framework domains. The framework supports process evaluation across three areas: examining implementation (including delivery, structure, fidelity, and reach), exploring mechanisms of impact (how the intervention works), and considering contextual factors influencing implementation

Please report categorical variables as counts and proportions n (%).

We have now revised the *Abstract: Results section* to report categorical variables as counts and proportions n(%).

***Pg 2 lines 19 – 21 Abstract/Results:** Of the 82 intervention clusters, 78 (95%) received intervention materials; 74 (90%) engaged with at least one activity and 15 (18%) in all activities.*

We have done the same throughout the manuscript when categorical variables.

Strengths and Limitations

P3L20-21: Consider if this is truly a strength or limitation of this study or a unique challenge that emerged from the trial context.

We agree that COVID-19 was a contextual challenge arising from the trial setting rather than an intrinsic strength or limitation of the study. We have therefore revised this bullet point to frame it as a strength of the intervention/implementation strategy, noting that the multi-modal intervention design (including digital components) supported at least some continuity of delivery and access when in-person engagement was constrained. We also updated the other bullet points to align with other revisions in the Methods section and to stay within the word count.

Page 4 Lines 8 - 10- Strengths and Limitations section
- The multi-modal intervention design, including digital components, supported access and some continuity of delivery when COVID-19 restricted in-person activities

Introduction

No comments.

Methods

P5L6: Please provide a reference to the FirstCPR trial indicating where it is described.
We have added a citation to the published FirstCPR trial paper(s) that describe the trial design and methods.

Please report categorical variables as counts and proportions n(%).
We have revised the reporting of categorical variables throughout the manuscript to present counts and proportions as n (%), in the Methods, Results, tables and supplementary materials.

Please provide measurements of variability for mean (standard deviation) and median (first quartile; third quartile). Please note that the interquartile range is technically a single value and is commonly confused with the first quartile and third quartile (Q1; Q3).
We have revised the manuscript to report measures of variability consistently for continuous variables. Means are now reported with standard deviations as mean (SD) for approximately normally distributed variables, and medians with minimum and maximum values, or the first (Q1) and third quartiles (Q3) or the interquartile range (IQR) for skewed data.

Page 8 Lines 19 - 22 Methods / Data Analysis: *Categorical variables are presented as counts and proportions, and continuous variables are summarised as means with standard deviations (SD) for approximately normally distributed variables and medians with minimum and maximum ranges or first (Q1) and third quartile (Q3) or interquartile range for skewed data.*

P7L5-11: I assume this is not a validated system. If this is correct, please consider discussing this limitation briefly in the Limitations section.

We agree that the implementation scoring system was specifically developed for this study and has not been formally validated. It was designed pragmatically to summarise intervention delivery and member uptake across intervention clusters based on available delivery and participation indicators and was used for descriptive comparison rather than as a validated measure of implementation or fidelity. We have now added a brief statement in the Limitations section acknowledging that the *scoring approach* is not validated which may limit comparability across settings and studies. We have also amended text in the Methods section to clarify this.

Pg 32 Lines 10 - 15 Limitations section: *The implementation scoring system used to summarise delivery and engagement across clusters was developed for this study and has not been formally validated. Thresholds were set pragmatically by the study team and may have introduced misclassification (e.g., organisations with different engagement patterns receiving similar scores). The score should therefore be interpreted as a descriptive summary of implementation variability, rather than a validated measure of fidelity or uptake.*

Pg 9 Lines 8-10 in Methods/Data Analysis section: *… This score was developed pragmatically (thresholds set by study team) for descriptive comparisons and was not intended or validated as a measure of implementation fidelity or intervention uptake.*

Results

The results section is lengthy which is an indication of the richness of the data that was collected. However, in its current state it is difficult to follow and at times the overall structure is confusing which risks that key findings are overlooked. Please consider whether the number and levels of subheadings can be reduced, whether all findings need to be discussed in this detail and please consider what the key findings are that need to be conveyed here and what information could also be made available in a supplementary results section. Additionally, it is currently difficult to distinguish between the various sources of information/data which makes interpretation challenging. I would suggest uniform wording to introduce findings from another source and/or separation within a paragraph. Please ensure that reporting of results is kept separate from discussion of results.

We appreciate the constructive feedback regarding the length, structure and clarity of the Results section. We agree that, in its original form, the level of detail and number of subheadings may have made it difficult to follow. To address this, the Results section has been substantially revised, words reduced where possible, fewer levels and subheadings, and overall restructured for ease of readability and to align with the Tables that complement the text with illustrative quotes. The text also clearly aligns with the study aims, and headings are named accordingly (organisation enrolment/participation; and implementation—reach, uptake and engagement). Also, I have used consistent wording, and the section has been revised so reporting remains descriptive and distinct from interpretation, with interpretive commentary

and broader implications now consolidated within the Discussion. We hope that these changes make it all more readable without losing the richness of the data.

Please see ***Page 10 for Results section -*** *now reorganised as below*

ORGANISATION ENROLMENT AND FACTORS INFLUENCING STUDY PARTICIPATION

Organisation screening, eligibility, and enrolment (quantitative, supported by Figure 2)

Barriers to organisation enrolment/participation

Enablers to organisation enrolment/participation

INTERVENTION IMPLEMENTATION (REACH, UPTAKE AND ENGAGEMENT)

Reach (distribution / exposure to intervention components)

Uptake (opt-in and in-person attendance)

Engagement (interaction with components and cluster-level variability

Participant feedback on intervention (acceptability and suggestions)

Factors influencing intervention implementation and member engagement (Table 2)

-**Contextual /extraneous factors**

Subthemes listed (bold and italic, no numbering)

-**Intervention design and delivery-related factors**

Subthemes listed (bold and italic, no numbering)

P9L2-5: Please consider discussing this in terms of limited generalizability within the limitations section of the manuscript.

We agree that limited generalisability is an important consideration given the recruitment and retention patterns observed in the study. In response, we have expanded the Limitations section to explicitly address the limitations in the representativeness of the enrolled sample and the possibility that attrition over the 12-month period may affect our observation of implementation patterns. We have acknowledged the absence of data from non-participating organisations and non-engaged members, which may have resulted in missing perspectives on barriers to participation.

Page 31 Line 19 -24; Pg 32 Lines 1 - 2 Limitations section: *There is also a risk of selection bias and limited generalisability as only 167 of 387 eligible organisations participated. Those who took part may have had greater capacity, motivation or leadership support for BLS training than those who declined or were not retained, limiting transferability of findings to time-poor organisations or those with limited facilitation capacity. Attrition over follow-up, with additional dropouts before the 12-month assessment, may also have influenced observed implementation patterns. In addition, there was limited input from non-participating organisations and from members who did not engage, which may have introduced bias, missing perspectives and an incomplete understanding of barriers to participation.*

P11L10: Please remain specific when describing reasons for withdrawal or loss to follow-up.

We have revised the Results to explicitly report reasons for withdrawal and to clarify that reasons for loss to follow-up could not be confirmed and mainly reflected non-response despite several attempts to contact the organisation committee.

Pg 14 Lines 2 - 11: Results section: *By 12 months, eight (15.6 %) intervention organisations withdrew, six of these were sports groups (Supplement Table S5). Reasons for withdrawals included limited committee capacity/time, competing operational priorities, or disruptions (e.g., flood damage, COVID-19-related restrictions, off-season), perceived limited benefit or low member interest (Supplementary Table S5). A greater number of control clusters withdrew (n=15, 18.1%), and an additional 28 (33.7%) were lost to follow-up. Common reasons for withdrawal were similar to those of the intervention group but also included a change in leadership with reduced interest. Reasons for loss to follow-up were largely unknown as organisations did not respond to contact attempts.*

P12L22-24: Please provide information on the population that responded to the survey and whether there is information on survey recall available. Please consider discussing the limitations and potential bias arising from voluntary surveys and potentially limited recall in the limitations section.

We have clarified the characteristics of respondents and we also now explicitly state that participants were asked to reflect on their recollection of CPR training activities over the preceding 12 months. In line with your recommendation, we have added text to the Limitations section, highlighting the potential for selection bias due to the survey's voluntary nature and the possibility of recall bias given the 12-month reporting window. These revisions acknowledge that feedback may not fully represent all participants.

Page 7 Lines 17 - 21 Methods/Data sources: *At the 12-month follow-up, participants in intervention clusters were invited to provide feedback on the intervention and its implementation via surveys and focus groups. Respondents were voluntary or opt-in, and survey items asked participants to reflect on their recollection of CPR training at their organisation over the preceding 12 months (See Supplement S2).*

Page 17 Lines 2 - 6 Results section: *Participant feedback at 12-month follow-up provided additional insight into the acceptability and perceived usefulness of the intervention components. In total, 189 participants from 46 intervention clusters (51% females, median age 62 years, 55% Australian-born) provided free-text feedback on intervention activities.*

Pages 33 Lines 4 - 9 Limitations section: …*12-month feedback was collected through voluntary surveys, which could introduce selection bias, for example, if respondents were more engaged with the intervention than non-respondents. As survey items required participants to recall activities conducted over the preceding year, responses may also be affected by recall bias. As a result, reported perceptions may not fully represent the broader*

intervention population, and estimates of acceptability or usefulness should be interpreted with caution.

P14L5: Please avoid the numbering of subheadings as this may lead to confusion with the two overall result sections.
We have now removed numbering across all subheadings in the Results section.

P14L18-19: I am unsure if this is speculation or results being reported. Please clarify this sentence and if needed move it to discussion sections.

We have revised the text to clarify that the observation was derived from a few participant accounts recorded in field notes. We retained it in the Results with explicit attribution and have noted that any effect on engagement was not quantified.

Pg 19 Lines 2 - 6 Results section: *Several text message scams occurred during the same period, and a few participants expressed hesitancy to open links in texts (field notes). Unfortunately, we were unable to quantify how much these events effected engagement with the intervention.*

P14L21-22 and P15L1-2: This seems to be interpretation or speculation. In any case, this would require data that can show this effect on participation levels. The current statement can be used as a speculation in the discussion but should still be supported by data.

The statements regarding competing priorities and seasonality were based on data (researcher field notes and liaison communications) collected during recruitment/implementation and are not speculative. We have revised the wording in the Results to explicitly attribute these observations to the data source. We also noted an illustrative example from the researcher's notes in Table 2 ("Not interested in participating anymore as we are in the off-season" – Football club, urban location). However, it was not possible to quantify the extent to which this factor affected overall participation levels.

Pg 19 Lines 8 – 12 Results section: *Liaisons cited competing organisational priorities such as ongoing community initiatives or seasonal sports schedules as influencing participation levels. Field notes and liaison communications indicated that seasonality constrained engagement in sports clubs, particularly during the off-season (Table 2).*

P15L6: The sentence seems to be more appropriate for a discussion section.
We revised the sentence in the Results subsection to remove interpretive framing and to ensure statements are descriptive.

Pg 19 Lines 18 - 21 Results section*: Interviews and field/researcher notes consistently highlighted the community liaison as a critical enabler and facilitator of implementation.*

Their commitment and time availability influenced the extent of delivery and member participation.

Since this is a key finding, we have further highlighted the liaison role and its implications in the ***Discussion section. See Page 28 Lines 1 - 3****.....and the pivotal role of organisation liaisons*

Discussion
P25L17-18: While it is fine to contextualize limitations, please avoid mentioning strengths in the limitations section.
This sentence has now been removed from the ***Limitations section*** see ***page 32 lines 15 -16*** of updated tracked manuscript.

Conclusions
The conclusions section is substantially too long, and I would suggest to the authors to focus on key aspects they wish to convey that are supported by the results of this study. Any other parts can be integrated into the discussion.

We have substantially shortened the ***Conclusions section (Page 32, now 147 words***) to focus only on the key take-home messages directly supported by the results of this process evaluation. The revised Conclusions are explicitly structured to reflect the study aims, and broader implications beyond the primary findings have been consolidated within the Discussion to improve clarity and maintain appropriate separation between interpretation and concluding statements.

I hope my comments help to further improve the authors' valuable work.

Many thanks, we are grateful for your constructive feedback. Your comments, along with those of the second reviewer, were indeed very helpful and have helped us strengthen this manuscript and improve its readability.

VERSION 2 - REVIEW

**Reviewer**	**1**
**Name**	**Myklebust, Helge**
**Affiliation**	**Laerdal Medical Cooperation, Strategic Research**

Date **16-Mar-2026**

COI

The authors have made major revision to the original manuscript, in line with the reviewer's suggestions. When reading again, it is striking to read that this large program seemed to have a rather small impact: Among 35 531 members in total, 742 participated in the 1h informal and educational session while 406 attended the 3h hands on CPR training. The authors state in the discussion that generating strong interest in the intervention proved challenging, (P23). The discussion also mention the importance of real world risk-context as motivating factors (most arrest happen at home, which means the lifesaver will be family or otherwise close to the victim). Is this the real problem? That the First CPR narrative speaks about "someone" as the victim is a stranger vs "a person you love". Should the narrative speak more about family safety and workplace safety (where we spend most of our time)? The authors may consider "exploring motivating factors among lay citizens regarding attending CPR training" as future research. Suggested reading: "Factors impeding dispatcher-assisted telephone cardiopulmonary resuscitation" (Samantha Hauff 2003) describes the relationship between lifesavers and patient. "A nationwide survey of first aid training and encounters in Norway" (Bakke 2017) shows the importance of workplace CPR training, which is further emphasized by " Contemporary levels of cardiopulmonary resuscitation training in Denmark" (Juul Grabmayr 2022).
